# Differential Intrahepatic Phospholipid Zonation in Simple Steatosis and Nonalcoholic Steatohepatitis

**DOI:** 10.1371/journal.pone.0057165

**Published:** 2013-02-25

**Authors:** Julia Wattacheril, Erin H. Seeley, Peggi Angel, Heidi Chen, Benjamin P. Bowen, Christian Lanciault, Richard M.Caprioli, Naji Abumrad, Charles Robb Flynn

**Affiliations:** 1 Columbia University, College of Physicians and Surgeons, Columbia University Medical Center, New York, New York, United States of America; 2 Mass Spectrometry Research Center, Vanderbilt University Medical Center, Nashville, Tennessee, United States of America; 3 Department of Biostatistics, Vanderbilt University Medical Center, Nashville, Tennessee, United States of America; 4 GTL Bioenergy and Structural Biology, Life Sciences Division, Lawrence Berkeley National Laboratory, Berkeley, California, United States of America; 5 Department of Pathology, Vanderbilt University Medical Center, Nashville, Tennessee, United States of America; 6 Department of Surgery, Vanderbilt University Medical Center, Nashville, Tennessee, United States of America; National Institute for Viral Disease Control and Prevention, CDC, China, China

## Abstract

Nonalcoholic fatty liver disease (NAFLD) occurs frequently in a setting of obesity, dyslipidemia and insulin resistance, but the etiology of the disease, particularly the events favoring progression to nonalcoholic steatohepatitis (NASH) as opposed to simple steatosis (SS), are not fully understood. Based on known zonation patterns in protein, glucose and lipid metabolism, coupled with evidence that phosphatidylcholine may play a role in NASH pathogenesis, we hypothesized that phospholipid zonation exists in liver and that specific phospholipid abundance and distribution may be associated with histologic disease. A survey of normal hepatic protein expression profiles in the Human Protein Atlas revealed pronounced zonation of enzymes involved in lipid utilization and storage, particularly those facilitating phosphatidylcholine (PC) metabolism. Immunohistochemistry of obese normal, SS and NASH liver specimens with anti-phosphatidylethanomine N-methyltransferase (PEMT) antibodies showed a progressive decrease in the zonal distribution of this PC biosynthetic enzyme. Phospholipid quantitation by liquid chromatography mass spectrometry (LC-MS) in hepatic extracts of Class III obese patients with increasing NAFLD severity revealed that most PC species with 32, 34 and 36 carbons as well as total PC abundance was decreased with SS and NASH. Matrix assisted laser desorption ionization - imaging mass spectrometry (MALDI-IMS) imaging revealed strong zonal distributions for 32, 34 and 36 carbon PCs in controls (minimal histologic findings) and SS that was lost in NASH specimens. Specific lipid species such as PC 34∶1 and PC 36∶2 best illustrated this phenomenon. These findings suggest that phospholipid zonation may be associated with the presence of an intrahepatic proinflammatory phenotype and thus have broad implications in the etiopathogenesis of NASH.

## Introduction

Nonalcoholic fatty liver disease (NAFLD) is the most common form of liver disease in Western countries with an estimated 80% of cases in the morbidly obese population and approximately 20–30% in the general population [1]. Hepatic fat accumulation in the absence of other liver diseases is manifest as simple steatosis (SS) in most individuals with NAFLD, although concomitant necroinflammatory changes may result in a minority (3–5%); these histologic features are termed nonalcoholic steatohepatitis (NASH). The relationship between steatosis and the pathogenesis of NASH is controversial, with data suggesting lipid quality and quantity may be important in the pathogenesis of NASH [2], [3]. Since pure overabundance of lipid is not sufficient for the development of NASH, it seems reasonable to hypothesize that a regional oversupply of a particularly noxious lipid species, or likewise the depletion of a protective lipid species, could have important mechanistic and clinical consequences. However, the identity and regional distribution of these lipid species, prior to speculation regarding their noxious or protective roles, must be described.

Hepatic lipid mobilization and storage is a highly dynamic and tightly regulated process influenced by physiologic, hormonal and nutrient cues. The dual supply of hepatic blood flow establishes structured environments, defined as zone 1 (periportal) to zone 2 (midzonal) to zone 3 (perivenular) within the liver acinus. This organization results in cellular adaptations manifest at enzymatic, metabolic and structural levels. Most extensively studied has been the zonation of enzymes facilitating carbohydrate, ammonia, glutamine and xenobiotic metabolism and it is generally appreciated that the zonation of lipid metabolism is much less pronounced [4], [5]. Most *in situ* information on the metabolic zonation of lipids comes from gene expression measurements of proteins regulating lipid metabolism such as acetyl-coA carboxylase [6], β-hydroxy-butyryl-CoA dehydrogenase [7], 3-hydroxy-3-methylglutaryl-CoA (HMG-CoA) synthase [8], and carnitine palmitoyltransferase I [6], [9]. Lipogenesis, inferred by increased acetyl-CoA carboxylase mass and activity measurements, occurs primarily in periportal (zone 1) hepatocytes [10]. Fatty acid oxidation, on the other hand, has been reported to occur preferentially in perivenular (zone 3) zones as suggested by the mildly increased expression of phosphatidate phosphatase and apolipoprotein C2 [6], [11], [12]. The distributional differences of specific lipid molecules within the liver lobule may bear strong associations with the metabolic and histologic changes observed between unique disease states as in SS and NASH. Few of these findings have been demonstrated directly in humans and little is known regarding how lipid zonation changes with disease, diet or environmental challenge.

**Figure 1:**
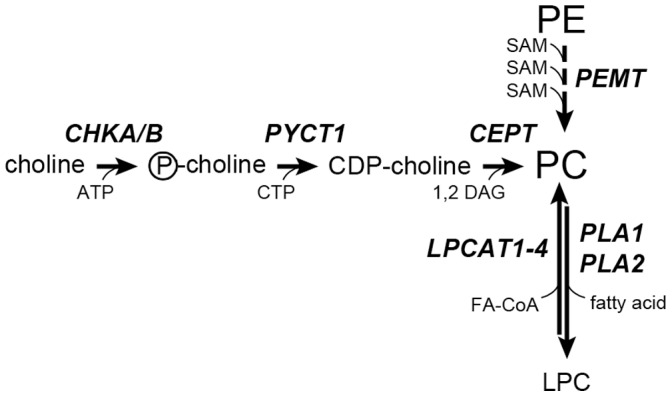
Schematic representation of major phosphatidylcholine (PC) biosynthetic pathways in human liver. Dietary choline is converted to phosphocholine by the actions of choline kinase (CHK), and then reacted with cytidine triphosphate (CTP) to form cytidine diphosphocholine (CDP-choline). Choline/ethanolaminephosphotransferase (CEPT) catalyzes the final step in PC biosynthesis by reacting *sn-*1,2-diacylglycerols (DAGs) with CDP-choline to form PCs. The second major pathway for PC biosynthesis involves three sequential methylation reactions of phosphatidylethanolamines (PE) with S-adenosylmethionine (SAM) by the actions of phosphatidylethanolamine *N-*methyltransferase (PEMT). The acyl distribution and composition of fatty acids (FAs) within each PC is continually modified by the actions of multiple phospholipases (primarily A1 and A2 isoforms) and lysophosphatidylcholine acyltransferases (LPCAT1-4). The PCs synthesized by either pathway are readily converted into TAG.

The most abundant lipids aberrantly stored in the liver in NAFLD are triglycerides (TAGs). Lesser known, but likewise partly composed of fatty acids are phospholipids. While TAG storage in the liver is associated with clinical consequences such as impaired glucose tolerance, little is known about other lipid fractions and disease development. Several studies have implicated changes in phosphatidylcholine (PC) species and abundance to be critical in promoting NASH. PCs account for as much as 65% of triglycerides in the normal murine liver [13] and evidence suggests that PC content is reduced in SS and NASH [14]. Furthermore, metabolism of PC species has been linked to NASH pathogenesis: hepatic deletion of phosphocholine cytidylyltransferase (PCYT1) and knockdown of LPCAT3, two enzymes involved in PC metabolism, results in marked reductions in VLDL secretion, a key factor in the development of NASH in humans [15]. PCs are synthesized in mammals by two predominant pathways (**Figure 1**). In the first pathway, CDP-choline, generated by the sequential actions of choline kinase (CHK) and phosphocholine cytidylyltransferase (CEPT) on dietary choline, reacts with *sn*-1,2 diacylglycerols (DAGs) to form PC [16], [17]. A second pathway, accounting for 30% of hepatic PCs, involves sequential methylation of phosphatidylethanolamine (PE) with S-adenosylmethionine catalyzed by the enzyme phosphatidylethanolamine *N-*methyltransferase (PEMT) [18], [19], [20]. The PCs synthesized by either pathway are readily converted into TAG.

Insight into the distribution of some hepatic lipid species was recently established by Debois et al. in normal and SS human liver specimens using cluster TOF-SIMS imaging [21]. They demonstrated periportal enrichment of α-tocopherol and cholesterol, along with a macrovesicular enrichment of TAGs, DAGs and FAs. However, the TOF-SIMS is a hard ionization technology resulting in significant lipid fragmentation, thus hampering the detection and discrimination of intact phospholipids. In the present study we investigated hepatic phospholipid abundance by quantitative lipidomic profiling and phospholipid localization by MALDI-IMS, a “soft” technique more conducive to intact lipid ionization. We determined the zonal distribution of various intact phospholipid species *in situ* in human liver specimens of control, SS and NASH using MALDI Imaging Mass Spectrometry (MALDI IMS). In addition, we examined the *in situ* hepatic localization of an important enzyme in the phosphatidylcholine biosynthetic pathway, PEMT, to investigate if altered zonation of PC biosynthetic enzymes may additionally contribute to NAFLD progression.

## Materials and Methods

### Ethics Statement

Subjects gave their informed written consent before participating in this study, which was approved by the Institutional Review Board of Vanderbilt University and registered at ClinicalTrials.gov (NCT00983463).

### Human Subjects

Class III obese women (n = 33, ages 26–59 years old) were recruited from the Center for Surgical Weight Loss at Vanderbilt University Medical Center prior to their scheduled bariatric procedures. Exclusion criteria included a history of previous liver disease (e.g. viral or autoimmune hepatitis, or hemochromatosis), significant alcohol use, concurrent infections, a cancer diagnosis within the previous 5 years, hemoglobin A1C >7.0, and the use of anti-diabetic drugs. All subjects underwent Roux-en-Y gastric bypass procedures. Wedge liver biopsies (200–1,000 mg) of the left lateral lobe were collected at the time of surgery. The tissue samples were immediately prepared and stored at *−*80°C for further histopathologic and mass spectroscopic analyses shown below.

### Reagents

Ammonium formate and lithium chloride were purchased from Sigma Chemicals (St. Louis, MO). Water, acetonitrile, 2-propanol, ethanol and methanol were purchased from EMD Chemicals (Gibbstown, NJ) and were of the highest analytical grade. 2,5-dihydroxybenzoic acid (DHB) was purchased from Acros Organics (Plans, NJ). Synthetic lipid standards were purchased from Avanti Polar Lipids (Alabaster, AL).

### Immunohistochemistry

Five-micrometer sections of formalin-fixed and paraffin-embedded liver tissue were baked at 60°C for 30 min, then de-paraffinized in xylene and hydrated in a graded ethanol to distilled water series. Antigen retrieval was performed in citrate buffer, pH 6.0 for 15 min. Slides were cooled then rinsed in distilled water and PBS, respectively, for 5 min. Endogenous peroxidases were blocked of endogenous peroxides with 0.3% hydrogen peroxide (Dako) for 20 min at RT prior to blocking overnight at 4°C in Protein Block. Sections were exposed to anti-PEMT antibody (HPA042375, Sigma-Aldrich, St. Louis, MO) diluted 1∶50 in Dako antibody diluent at 4°C overnight. PBS-washed sections were subsequently incubated with alkaline-phosphatase conjugated secondary antibody for 15 min at RT prior to development with chromagen substrate. Sections were counterstained with hematoxylin for 30 sec, prior to dehydration in 75% ETOH for 5 min.

### Lipid Quantification by HPLC ESI-MS

We utilized a MS^e^ rapid profiling screening strategy that enabled lipid quantifications in a total time of 18 min [22], [23]. Lipids extracted from liver specimens were resolved by HPLC and eluting peaks were analyzed by collision induced dissociation (CID) in a tandem quadrupole time-of-flight (Q-TOF) mass spectrometer (MS). This acquisition strategy permitted the untargeted identification of PC and PE species (**Figures S1, S2**) [23]. Lipids are denoted by a simplified nomenclature wherein the number of carbons and double-bonds in the side-chains are designated. For example, PC 36∶2 refers to a PC species with a total of 36 carbons in the 2 acyl chains and the total number of double bonds in the two acyl chains is 2; the assignment does not delineate side-chain species. Lipid identifications were made upon querying masses observed in precursor and matching fragmentary ion spectra against a predefined database using software packaged with the instrument. Lipid quantification relied on area under the curve (AUC) measurements of extracted ion chromatograms generated from precursor ion scan information.

Phosphatidylcholines were discriminated based on the presence of *m/z* 184.07 product ions corresponding to a phosphocholine polar head group [C_5_H_15_NPO_4_]^+^. PEs were discriminated based on spectra containing product ions of a phosphoethanolamine head group ([M+H*−*140]^+^ and *m/z* 186.22) (**Table S1**). Detailed description of lipid extraction is provided in **Methods S1**.

### MALDI Imaging Mass Spectrometry (MALDI IMS) Data Acquisition and Analysis

For each specimen, tissue substructures were first established in co-registered H&E-stained cryosections serial to those analyzed by MALDI IMS. Regions of interest identified in DHB-sublimated sections were subsequently sampled at 20 µm spatial resolutions using a TOF mass spectrometer equipped with a MALDI source. From a third set of serial sections, mass spectra were collected at 50 µm spatial resolution using Fourier transformed ion cyclotron resonance (FT-ICR) MALDI-IMS to establish species accurate mass. From the mass spectra acquired at each pixel position in both datasets, the abundance of an ion of interest was recorded and colored heat maps for regions within each liver specimen were generated.

Liver biopsies warmed to *−*20°C were mounted on cryostat chucks and then sectioned with a Leica CM 1900 cryotome. Twelve micron-thick sections of human livers were thaw-mounted onto gold-coated MALDI target plates. The organic matrix, 2, 5-dihydroxybenzoic acid (DHB) was applied to liver sections [24]. Serial sections were collected on glass slides and stained with hematoxylin and eosin (H&E). Magnified photomicrographs of each H&E stained liver section were obtained after scanning the slides using a Mirax Desk microscope slide scanner (Zeiss, Thornwood, NY) at a pixel resolution of 0.23 µm. Digital photomicrographs were exported using the Mirax Viewer software and used in FlexImaging as a registration image.

A MALDI TOF mass spectrometer with reflectron geometry (AutoFlex Speed, Bruker Daltonics, Billerica, MA) equipped with a SmartBeam laser (Nd:YAG, 355 nm) was operated in positive ion mode to acquire spectra data across 2×2 mm regions of all liver specimens. Full scan mass spectra were collected between 400 and 1800 *m/z.* Lipid images were acquired at 20 µm pixel size (spatial resolution), averaging 80 laser shots per pixel. [25] Images were visualized using BioMap software (3.8.0.3, Novartis, Basel Switzerland). Raw spectra from hepatic zones identified in each image were extracted and pre-processed using a modified Wave-spec package [26].

#### Calibration

All spectra were individually calibrated against abundant lipids (PC 34∶2 [M+K] – *m/z* 796.52; PC 34∶2 [M+H] – *m/z* 758.56; PC 36∶2 [M+K] – *m/z* 824.55; PC 38∶4 [M+K] – *m/z* 848.55; DAG-O 34∶4 [M+H] – *m/z* 575.50) unifying the *m/z* scales. Spectra were denoised using undecimated discrete wavelet transformation with hard thresholds empirically determined through a feedback loop [26], [27]. Spectra were normalized by the total ion current (TIC) method to enforce the constraint of equal TIC for each spectrum in the dataset [28]. AUCs were derived from the contents of each bin and these values were transformed onto log10 scale for the normality assumption of statistical analysis. Analysis of variance (ANOVA) with Bonferroni correction for multiple comparisons was used to compare differences in intensities across normal, SS and NASH groups.

## Results

### Anthropomorphic Measurements

The presence of steatosis, ballooning, inflammation and fibrosis was determined by a hepatopathologist using two independent histologic specimens for classification and subsequent scoring by the NAFLD Activity Score (NAS) [29]. This resulted in categorizing the obese subjects into three cohorts (n = 11 in each): (a) a cohort of subjects with normal histopathologic findings (referred to as “normal”); (b) a cohort with non-NASH NAFL (referred to as simple steatosis or “SS”), and (c) a group with established NASH histology.

Given the difficulties in obtaining sufficient material for both quantitative LC-MS/MS and MALDI IMS analyses we elected to study small, but well defined cohorts (**Table 1**). Cohorts (n = 8 in each) were similar in terms of ethnicity, BMI, fasting blood glucose and weight. Subjects with NASH were slightly older than the obese normal (mean ± SD: 48.5±2.1 in NASH vs. 40.6±2.1 in obese normal; *P*<0.01). Liver biochemistries including AST, ALT, AST/ALT ratios, bilirubin, albumin and platelets were not different among the three groups, however alkaline phosphatase levels (U/I) were elevated (*P*<0.01) by nearly two-fold in both SS (98.7±9.1) and NASH (92.2±5.9) vs. the obese normal (53.2±8.8). Obese normal specimens were almost uniformly void of steatosis, inflammation and fibrosis in comparison to both SS and NASH specimens. The mean NAS was highest (5.4±0.3) for NASH specimens (*P*<0.001 vs. SS or obese normal).

**Table 1:** Subject Characteristics.

n	11	11	11
Mean age (years)	40.6±2.1	32.7±1.8	48.5±2.1^a^
Gender (F/M)	11/0	11/0	11/0
Caucasian/African American	9/2	9/2	11/0
Weight (kg)	125.7±5.1	123.4±6.1	116.7±4.1
BMI (kg/m^2^)	46.4±1.9	46.2±2.2	45.2±1.8
Glucose (mg/dl)	93.6±3.8	115.1±11.8	108.3±10.7
AST (normal range: 0–65 U/I)	20.5±1.8	20.9±4.6	28.8±7.7
ALT (normal range: 0–65 U/I)	22.7±2.8	24.4±6.9	42.0±17.9
Alkaline phosphatase (U/I)	53.2±8.8	98.7±9.1 b	92.2±5.9^b^
AST/ALT ratio	0.98±0.08	1.01±0.14	0.88±0.07
Total bilirubin (mg/dl)	0.6±0.1	0.4±0.0	0.8±0.3
Albumin (g/dl)	4.1±0.1	4.1±0.1	4.1±0.1
Platelets (1000/mm^3^)	266.1±21.8	288.3±17.0	275.4±14.9
Steatosis (0–3)	0.1±0.1	1.5±0.2^c^	2.1±0.2^c^
Ballooning (0–3)	0.4±0.2	0.5±0.2	1.7±0.1^ab^
Inflammation (0–3)	0.1±0.1	0.1±0.1	1.5±0.2^cd^
Fibrosis (0–2)	0.1±0.1	0.2±0.1	0.6±0.2
NAFLD Activity Score (0–8)	0.5±0.2	1.8±0.3	5.4±0.3^cd^

Data presented as mean ± SEM. ALT, alanine aminotransferase; AST, aspartate aminotransferase, BMI, body mass index; SS, simple steatosis; NASH, nonalcoholic steatohepatitis.

a *P*<0.01 vs. SS;

b *P*<0.01 vs. normal;

c *P*<0.001 vs. normal;

d *P*<0.001 vs. NAFL (Kruskal-Wallace analysis of variance with Dunn’s post-test).

### Histopathologic Assessment of NAFLD in Wedge Liver Biopsies

Traditional histology-based assessments of H&E stained slides revealed hallmark patterns of injury in NASH specimens that were clearly different from the frank steatosis that helps to delineate lobules with SS and strikingly dissimilar from the uniform and hexagon portal tract arrangements that define obese normal specimens. In contrast to obese normal specimens that were largely devoid of visible micro- or macrosteatosis, SS specimens displayed numerous large lipid droplets, most prominent in hepatocytes surrounding the central vein (zone 3 hepatocytes). Portal tracts, comprised of bile ducts, hepatic arteries and portal veins were arguably the most identifiable features in all specimens. In SS and NASH, zone 1 hepatocytes surrounding portal tracts typically displayed less steatosis and tended to be mononucleate. In NASH samples, steatosis was accompanied by hepatocyte ballooning and inflammation most evident in zone 3, although these defining characteristics were also observed on occasion in zone 1.

### Measurements of Relative Abundance of Hepatic Phospholipids

**Figure 2:**
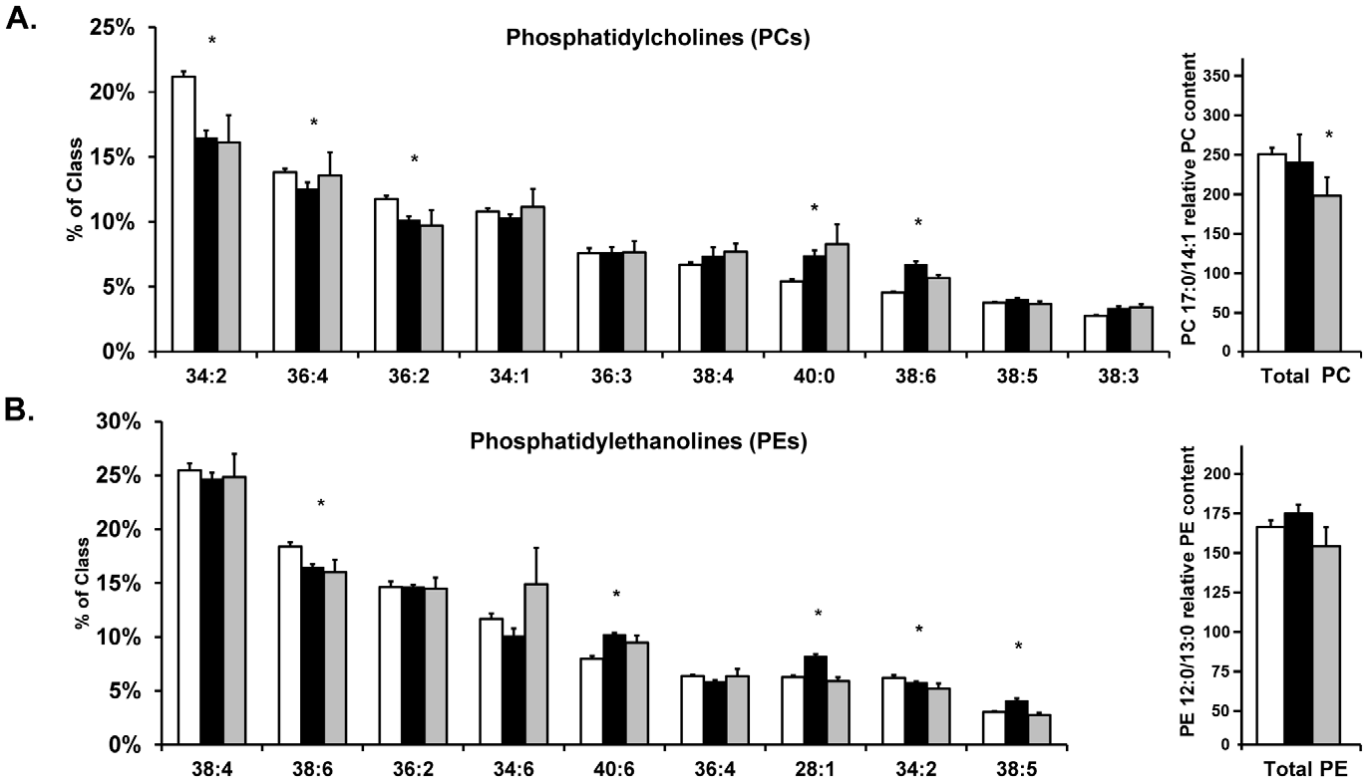
Comparison of lipid molecular species abundance by nano-LC MS. Hepatic lipid composition in controls (white), SS (black) and NASH (gray), for individual lipid species or for total PC or PE mass (far right) for (**A**) PC and (**B**) PE, respectively; n = 8 individuals per group. PEs are included here given their role as substrates for PC biosynthesis as mentioned in Figure 1. Only the most abundant lipids per class are shown (left). Total PC was normalized to the internal standard PC 17∶0/14∶1 while total PE was normalized to PE 12∶0/13∶0 (right). All values are expressed as the mean ± SEM. Testing of the null hypothesis by 1-way ANOVA with Bonferroni correction for multiple comparisons between cohorts is denoted by * *P*<0.05.

PCs are a class of phospholipids particularly amenable to analysis by ESI given their abundance and ease of ionization [30]. A comprehensive list of all lipids identified is available in **Table S2**. The most abundant PCs in all specimens, accounting for nearly half of all PCs detected, were PC 34∶1 (*m/z* 860.58), PC 34∶2 (*m/z* 758.56), PC 36∶2 (*m/z* 786.60) and PC 36∶4 (*m/z* 782.57). Nine of the nineteen quantified PCs (all [M+H^+^] adducts) were significantly different (*P*<0.05) among the three cohorts (**Figure 2A**). The most distinct differences in lipid species of the PC class were PC 34∶2 (*m/z* 758.56) and PC 36∶2 (*m/z* 786.60), which were decreased in both SS and NASH, and PC 40∶0 (*m/z* 846.70) which was significantly increased in SS and NASH compared to obese normal. Others such as PC 36∶4 (*m/z* 782.57) and PC 34∶1 (*m/z* 760.58) were decreased in SS and increased in NASH relative to obese normals. Lesser abundant PCs (≤10% of class) exhibited different patterns. PC 38∶4 (*m/z* 810.60), PC 40∶0 (*m/z* 846.70) and PC 38∶3 (*m/z* 812.61) were greater in SS and also increased in NASH. When considered in aggregate, total PC mass was significantly reduced in SS and NASH compared to controls.

Several PEs are found in human liver in minor quantities [14], [31]. Four PEs, all [M+H^+^] species, comprised the majority of hepatic PE content by class; PE 38∶4 (*m/z* 768.55), PE 38∶6 (*m/z* 764.52), PE 36∶2 (*m/z* 744.55) and PE 34∶6 (*m/z* 708.47) represented ∼58% of the PEs detected. Five of the nine identified species (indicated by asterisks in **Figure 2B**) were significantly different among cohorts, and all were decreased in NASH compared to SS specimens. Only PE 40∶6 (*m/z* 792.55) was increased in SS and NASH compared to obese normal controls. For certain PEs such as PE 38∶6 (*m/z* 764.52) and PE 34∶2 (*m/z* 716.52), species abundance decreased with disease severity. In the case of a few PE species, such as PE 40∶6 and PE 28∶1, SS specimen possessed a greater PE species content than did NASH or obese normal specimens. Total PE mass was not significantly different among cohorts.

### Phospholipid Zonation Analysis by MALDI IMS

**Figure 3:**
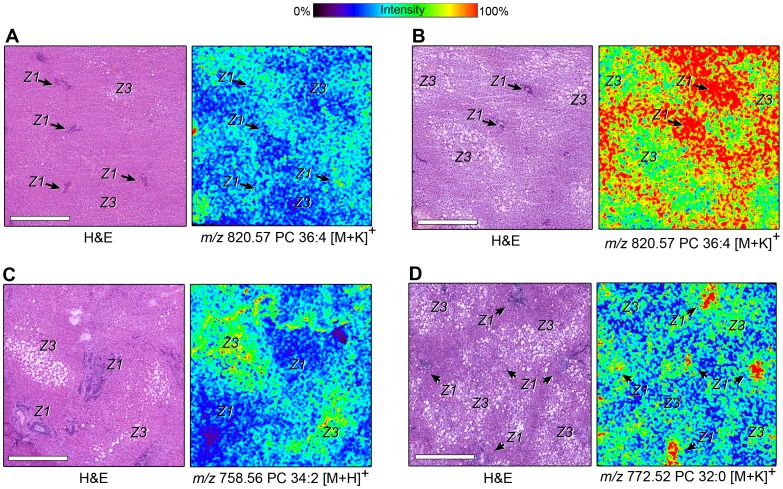
Multiple phospholipid distribution patterns in human liver as revealed by MALDI IMS. Representative photomicrographs of H&E sections (left panels, **A–D**) are marked to indicate locations of zone 1 (Z1) or zone 3 (Z3) hepatocytes. MALDI IMS images (right panels, **A–D**) of *m/z* 820.57, corresponding to PC 36∶4 [M+K]^+^, in (**A**) obese normal and (**B**) SS specimens, *m/z* 758.56, corresponding to PC 34∶2 [M+H]^+^ in SS specimens (**C**), and *m/z* 772.52, corresponding to PC 32∶0 [M+K]^+^ in (**D**) NASH specimens reveal distinct phospholipid patterning. An ion intensity color scale applicable to all images is shown at the top of figure. Scale bar = 500 µm.

MALDI revealed a remarkable diversity of PCs identified across the spectrum of all liver specimens with localizations ranging from diffuse to conspicuous depending upon the lipid molecular species. It is known that MALDI IMS using DHB as a sublimation matrix yields predominantly alkali metal adducts [M+Na]^+^ and [M+K]^+^ as well as lesser [M+H]^+^ species [32], [33]. Four general patterns characterized lipid species distributions (**Figure 3** **)**. The first pattern was azonal where ion intensities for a particular lipid varied little across the acinus. H&E photomicrographs and MALDI IMS images of an obese normal specimen, shown in **Figures 3A** **,** designate zone 1 (arrows) and zone 3 hepatocytes where *m/z* 820.56 PC 36∶4 [M+H]^+^ intensities were relatively similar. A second lipid distribution pattern, one characterized by increased zone 1 vs. zone 3 intensities, is illustrated by *m/z* 820.57 PC 36∶4 [M+K]^+^ in **Figures 3B** for a SS specimen. A third pattern, describes lipid ion intensities greatest in zone 3 hepatocytes surrounding the central vein, was most often observed in SS but not NASH or obese normal specimens. MALDI images for *m/z* 758.56 PC 34∶2 [M+H]^+^ best illustrate this pattern (**Figure 3C**). A fourth major pattern, one where ion intensities were robust over portal tracts alone (**Figure 3D** and **Figure S4**), was observed in all specimens irrespective of pathologic classification and was unique for *m/z* 772.53 PC 32∶0 [M+K]^+.^ A listing of all lipids identified by MALDI IMS is provided in **Table 2**.

**Table 2:** Lipid Molecular Species Identified in Human Livers by MALDI IMS.

Lipid	FA side chains (major^†^, minor^‡^)	Possible Species	*m/z* observed	*m/z* theoretical	Ion	FT-ICRΔm (ppm)
PC 32∶0	16∶0, 16∶0	PC 16∶0/16∶0	756.5516	756.5514	[M+Na]^+^	−3.23
			772.5258	772.5253	[M+K]^+^	−6.30
PC 34∶1	16∶0, 18∶1	PC 16∶0/18∶1	760.5854	760.5851	[M+H]^+^	−4.58
			782.5671	782.5670	[M+Na]^+^	−1.07
			798.5407	798.5410	[M+K]^+^	3.29
PC 34∶2	16∶0, 18∶2	PC 16∶0/18∶2	758.5694	758.5694	[M+H]^+^	0.00
			780.5518	780.5519	[M+Na]^+^	1.60
			796.5251	796.5259	[M+K]^+^	9.69
PC 36∶1	18∶0^†^, 20∶1	PC 18∶0/18∶1;	788.6165	788.6164	[M+H]^+^	−1.50
		PC 16∶0/20∶1	810.5828	810.5827	[M+Na]^+^	−1.16
			826.5723	826.5723	[M+K]^+^	−0.44
PC 36∶2	18∶0^†^, 18∶2^‡^,	PC 18∶0/18∶2;	786.6012	786.6007	[M+H]^+^	−6.46
	16∶0^†^	PC 16∶0/20∶2	808.5828	808.5827	[M+Na]^+^	−1.16
			824.5566	824.5566	[M+K]^+^	0.17
PC 36∶3	18∶1^†^, 16∶0^†^,	PC 18∶1/18∶2;	784.5854	784.5851	[M+H]^+^	−4.56
	18∶2^‡^, 20∶3^‡^	PC 16∶0/20∶3	806.5671	806.5670	[M+Na]^+^	−1.41
			822.5412	822.5410	[M+K]^+^	−2.88
PC 36∶4	16∶0, 20∶4	PC 16∶0/20∶4	782.5690	782.5694	[M+H]^+^	5.78
			804.5515	804.5514	[M+Na]^+^	−2.04
			820.5251	820.5253	[M+K]^+^	2.59
PC 38∶3	18∶3, 16∶1	PC 16∶1/22∶2;	850.5729	850.5723	[M+K]^+^	−7.48
		PC 18∶3/20∶0				
PC 38∶4	18∶0, 20∶4		848.5564	848.5566	[M+K]^+^	2.52
PC 38∶5	18∶3, 16∶0	PC 18∶3/20∶2;	846.5411	846.5410	[M+K]^+^	−1.62
		PC 16∶0/22∶5				
PC 38∶6	16∶0, 22∶6	PC 16∶0/22∶6	844.5261	844.5253	[M+K]^+^	−8.85
PA 32∶0	14∶0^†^, 10∶0^†^	PA 14∶0/18∶0	649.5170	649.5167	[M+H]^+^	−5.88
		PA 10∶0/22∶0				
PA 38∶3	18∶3, 20∶0	PA 18∶3/20∶0	749.5092	749.5092	[M+Na]^+^	−0.31
PA 38∶5	16∶1^†^, 18∶3^‡^,	PA 18∶3/20∶2	761.4522	761.4518	[M+K]^+^	−5.52
	20∶2^‡^	PA 16∶1/22∶4				
PA 38∶6	16∶0, 18∶2	PA 16∶0/22∶6	759.4369	759.4362	[M+K]^+^	−9.20
		PA 18∶2/20∶4				
PA 40∶4	18∶3, 22∶1	PA 18∶3/22∶1	775.5248	775.5248	[M+Na]^+^	−0.29
PA 40∶5	18∶1, 22∶4	PA 18∶1/22∶4	773.5097	773.5097	[M+Na]^+^	0.00
			789.4825	789.4831	[M+K]^+^	7.78
PA 44∶6	22∶1, 22∶5	PA 22∶1/22∶5	827.5574	827.5561	[M+Na]^+^	−15.4

Abbreviations: PC − phosphatidylcholine, PA – phosphatidic acid, FT-ICR – Fourier transformed ion cyclotron resonance MS, m/z - mass/charge.

**Figure 4:**
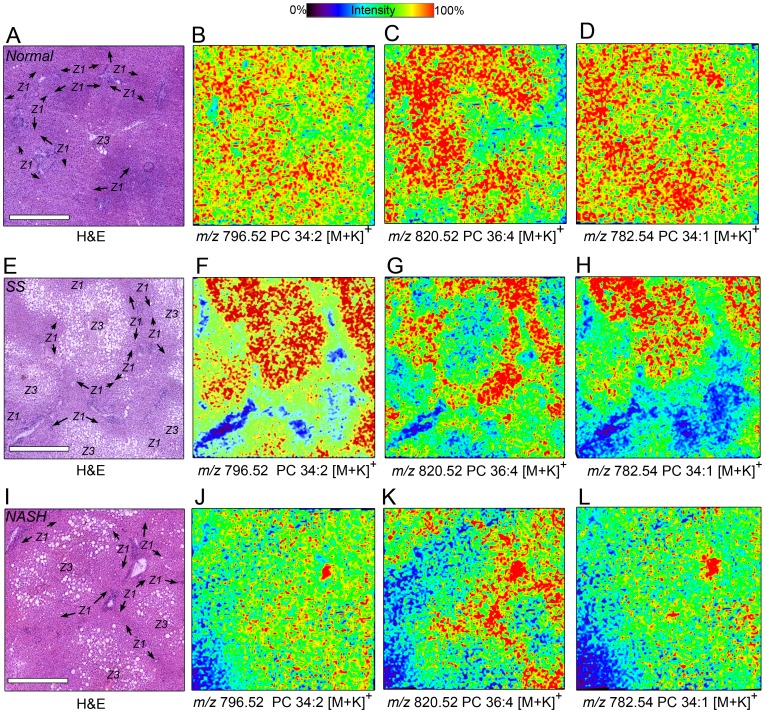
Lipid MALDI ion images reveal differences in PC zonation across the liver lobule. Representative MALDI IMS images of selected liver specimens obtained from obese subjects with normal liver histology (**A–D**), SS (**E–H**), and NASH (**I–L**). (**A, E, I**) Photomicrographs of H&E stained sections from each specimen (far left column) indicate lobular zones (zone 1 - Z1; zone 3 - Z3). Comparison of selected PCs by disease state: MALDI images of *m/*z 796.52 PC 34∶2 [M+K]^+^ in obese normal (**B**), SS (**F**), and NASH (**J**) specimens. MALDI images of *m/*z 820.52 PC 36∶4 [M+K]^+^ in obese normal (**C**), SS (**G**), and NASH (**K**) specimens. MALDI image of *m/*z 782.54 PC 34∶1 [M+K]^+^ in obese normal (**D**), SS (**H**), and NASH (**L)** specimens. Ion intensity color scale for all ion images is shown at the top of panels B and C. Scale bar = 500 µm.

In liver specimens obtained from histologically normal obese subjects (exemplified in **Figure 4A–D**), the most abundant species observed both by HPLC-ESI-MS/MS and MALDI IMS, namely *m/z* 796.52 PC 34∶2 [M+K]^+^, displayed an azonal distribution (pattern 1; **Figure 4B**). The second most abundant PC in normal obese subjects, *m/z* 820.52 PC 36∶4 [M+K]^+^, consistently displayed a zone 1 enriched distribution (pattern 2; **Figure 4C**). Another lipid expression pattern emerged, that of *m/z* 782.54 PC 34∶1 [M+K]^+^, showed limited zone 1 accumulations (pattern 3; **Figure 4D** **)**. In the SS cohort (exemplified in panels **Figure 4E–H**), PC 34∶2 [M+K]^+^ showed zonation different from that observed in obese normal subjects; distributions were zone 3 dominant (pattern 3, **Figure 4F**). Interestingly, PC 36∶4 [M+K]^+^, which accumulated almost exclusively in zone 1 (pattern 1; **Figure 4G** **)** displayed similar zonation to its histologically normal counterpart. The zonation of PCs with the linoleate fatty acid replaced by oleate, e.g. PC 34∶1 [M+K]^+^ (pattern 2; **Figure 4H** **),** was similar to their less saturated counterparts (PC 34∶2 [M+K]^+^ shown in **Figure 4F**). In NASH specimens (**Figure 4I–L**) the detected phospholipid distributions were mostly azonal, as illustrated by PC 34∶2 [M+K]^+^, (pattern 1; **Figure 4J** **)** and PC 34∶1 [M+K]^+^, **3L** (pattern 1). Similar to SS specimens, NASH specimens displayed strong zone 1 intensities for PC 36∶4 relative to zone 3 (**Figure 4K**). The zonation of most other lipids was minor and there was no discernible correlation between the respective pattern of lipid distribution and NAS score in NASH specimens.

### Immunohistochemistry of PEMT in Obese Human Livers

To understand some of these observed zonal differences between lipid-rich states, we queried the human protein atlas portal (www.proteinatlas.org) for 87 different proteins described to be involved with phospholipid, fatty acid and triglyceride metabolism (**Table S3**). These proteins were localized *in situ* in normal human liver tissue with 99 different antibodies in a 54 year old female. We identified pronounced zonation for at least 16 different enzymes mediating lipid metabolism (**Figure S3**) and mild zonation for at least 12 others. In this histologically normal liver (M-0100), strong perivenular staining was observed for carnitine palmitoyltransferase 1B (CPT1B; **Figure** **S3A**), carnitine acyltransferase (CRAT; **Figure** **S3B**) and the carnitine/acylcarnitine transporter (SLC25A20; **Figure** **S3C**), all facilitating fatty acid transport. Zonation was similarly observed for the mitochondrial fatty acid oxidation enzymes, acyl-CoA dehydrogenase and very long chain (ACADVL; S**3D**) and 2,4-dienoyl CoA reductase 1 (DECR1; **S3E**), the cytoplasmic enzymes acetyl-CoA carboxylase (ACACB; **Figure** **S3F**) and diacylglycerol O-acyltransferase 2 (DGAT2; **Figure** **S3G**) as well as the lipid droplet associated protein perilipin 1(PLIN1; **Figure** **S3H**). In particular**,** several proteins facilitating phospholipid metabolism also displayed zonal expression. Preferential perivenular (zone 3) zonation was clearly evident for choline kinase α (CHKA; **Figure** **S3I**), phosphocholine cytidylyltransferase 1 (PYCT1A; **Figure** **S3J**), phosphatidylethanolamine *N*-methyltransferase (PEMT; **Figure** **S3K**), glycerol-3-phosphate acyltransferase 2 (GPAT2; **S3M**) as well as the phospholipases A2 G15, A2 G4F and B1(PLA2G15, PLA2G4F and PLA2B1 **Figure** **S3N-S3P**, respectively). Only phosphocholine cytidylyltransferase 2 (PCYT2; **Figure** **S3L**) displayed a perivenular dominant (zone 1 to zone 3) expression pattern.

**Figure 5:**
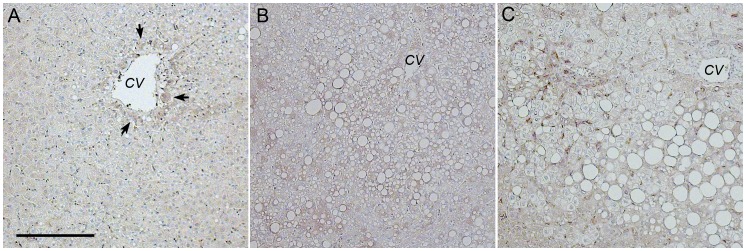
Phosphatidylethanolamine methyltransferase (PEMT) localization differs with progression of NAFLD. Monoclonal antibodies were used to localize PEMT in liver specimens of bariatric subjects sampled intra-operatively at the time of bariatric surgery. (**A**) The *in situ* localization of PEMT in an obese normal control subjects was primarily in hepatocytes surrounding the central vein (zone 3; black arrows). (**B**) PEMT in a SS specimen was localized throughout the liver acinus in zones 1–3. (**C**) PEMT in NASH specimen accumulated at sites of inflammation (white arrows), as well as in zones 1–3.

To gain insight into what, if any, changes may exist with regard to PC biosynthetic enzymes commensurate with NAFLD progression, we examined *in situ* the localization of PEMT, a primary PC biosynthetic enzyme. **Figure 5A** illustrates the strong perivenular localization of this enzyme in a histologically normal obese liver. This contrasts with panlobular distribution of the enzyme in SS (**Figure 5B**) and NASH, and with notable localization in necroinflammatory sites in NASH specimens (**Figure 5C**).

## Discussion

Aberrant hepatic lipid metabolism has long been proposed to be central in the pathogenesis of NASH, but the exact mechanism(s) underlying necroinflammatory changes on background steatosis remain to be fully understood. The differential abundance of phospholipids, particularly PCs and PEs, has been studied quantitatively but these studies have largely been either in mice [34], [35], [36], [37], in anthropomorphically-diverse cohorts [30], or with destructive analytical methods such as methylation or acid/alkaline digestion that provide information on fatty acid composition within a lipid class but do not identify a specific PC [31], [38], [39]. The current study identifies, quantifies and localizes specific choline-containing lipids in human liver and reveals a previously unappreciated zonation of specific molecular PCs that are either lost or preserved in association with NAFLD severity. Given these ostensibly protein-mediated changes, we further demonstrated the zonation pattern of one hallmark PC metabolic enzyme, PEMT.

PCs account for nearly two-thirds of TAGs in liver [13] During normal conditions, the liver is the predominant site of PC biosynthesis; reductions in intra-hepatic and circulating PC levels, either by genetic [34], [35] or dietary means [36], [37], stimulates nascent VLDL particle recycling with simultaneous compensatory hepatic uptake of circulating PC-poor VLDL. Both phenomena have been described to occur with NASH. Our findings that many PCs such as PC 34∶2, PC 36∶2, PC 38∶4, PC 40∶0 and PC 38∶3 showed altered abundance with increasing histologic severity while others (PC 34∶1, PC 36∶3. PC 38∶5) did not suggest species-specific effects (**Figure 2A**). Thirty-six and 34 carbon PCs were the most abundant and total PC mass was reduced in both SS and NASH compared to controls. PE content also was changed in a species specific manner, however trends in abundance changes were subtle and most significant differences were in lesser abundant PEs.

Similar to our observations, other investigators have also found significant alterations in lipid abundance with NAFLD [31]; they differ, however, in the subclasses with detectable differences in disease states. Allard *et al.* demonstrated significant reductions in *n−6* and *n−3* polyunsaturated fatty acids (PUFAs) in SS and NASH in Class I obese subjects [38]. Araya *et al.* found that the fatty acid composition of liver phospholipids in a cohort of Class III obese subjects had similar proportions of total PUFAs and monounsaturated (MUFAs) in SS and NASH [39]. Puri *et al.* detected significant decreases in total PC content in Class II obese subjects (BMI = 35–38 kg/m^2^) and demonstrated decreased eicosapentanoic acid (20∶5*n−3*) and docosahexanoic acid (22∶6*n−3*) in hepatic TAGs in NASH [14]. However, in each of these studies the destructive analysis of PCs permitted compositional determinations of fatty acids cleaved from the PC glycerol backbone, but precluded determination of intact PCs.

Hepatic zonation was first described in 1963 by Elias *et al* [40]. This was based on observations of functional hepatocyte heterogeneity along the porto-central axis of the liver-cell plate. This concept, referred to as “metabolic zonation”, was later refined by Katz and Jungermann [41] and proposed to explain how differing and occasionally opposing functions could coexist in the liver [42]. Thus far, several studies have identified metabolic zonation for glucose metabolism [43], ammonia detoxification [44], [45], [46] and metabolism of drugs and xenobiotics [5]. However, there is lesser and more conflicted information, related to hepatic zonation of lipids [4], [5]. Using MALDI-IMS we observed zonal distributions for several different PC species in liver specimens identified as histologically normal, SS or NASH.

In conjunction with the altered phospholipid distributions, we also identified *in situ* differential zonation and abundance of an important enzyme governing PC biosynthesis, PEMT. In SS, increased zone 3 steatosis was coincident with a redistribution of PEMT from perivenular sites to that of a non-descript panlobular pattern. With NASH, the panlobular distribution of PEMT pervaded and was accompanied by additional localized expressions at necroinflammatory sites. This loss and, at times, reverse zonation of lipids appears strongly linked to the disarray of enzymes related to PC biosynthesis. These microenvironmental changes suggest enzymatic differences in localization with potential alterations in function.

**Figure 6:**
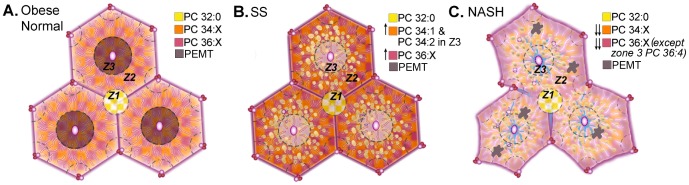
Diagram depicting loss of phospholipid zonation in NASH. (**A**) In obese normal liver tissue the PCs with 34 and 36 carbons, were most abundant and localized to zones 2 (Z2) and 3 (Z3). PC 32∶0 localized exclusively to portal tracts in all specimens. PEMT localized exclusively to zone 3 hepatocytes. (**B**) In SS specimens, there were increased amounts of PC 34 and 36 carbon PCs in zone 3 and zone 2 but a decrease in their abundance in zone 1. (**C**) In NASH, the zonation of most lipid molecular species was reduced with few exceptions. In both SS and NASH, PEMT expressions were panlobular, but with NASH, additional staining was observed at sites of inflammation.

A summary of our findings related to the changes in abundance and mass of PCs in relation to disease severity is provided in **Figure 6**. In normal obese liver, 36 and 34 carbon PCs are most abundant and localized primarily to zones 2 and 3 (**Figure 6A**). PEMT expression (brown) was greatest in hepatocytes surrounding the central vein. PC 32∶0 expression was robust at portal triads (Z1, yellow and white checkered area). In SS, zone 3 accumulation of these 34:x and 36:x PCs, especially PC 34∶1 and PC 34∶2, was increased (**Figure 6B**). In NASH, the intrahepatic zonation of PC 34∶1 and PC 34∶2 was lost, but that for PC 36∶4 (zone 1> zone 3) was preserved. The strong zonation of PEMT in perivenular hepatocytes was lost in SS and NASH (**Figure 6C**).

Our findings are from a rather small cohort of morbidly obese subjects that are representative for one of the dominant, but not only, groups at risk for developing NASH. Some of the more subtle zonation patterns we observed in normal, SS and NASH specimens may require profiling a larger number of specimens to establish clarify lipid specific and protein-mediated effects with regards to aberrant lipid storage and metabolism.

## Supporting Information

Figure S1 **Calibration curves for lipid standards were determined at the concentration range indicated.** Increasing amounts of **A**) 1-heptadecanoyl-2-(9Z-tetradecenoyl)-sn-glycero-3-phosphocholine (PC 17∶0–14∶1) and **B**) 1-dodecanoyl-2-tridecanoyl-sn-glycero-3-phosphoethanolamine (PE 12∶0–13∶0) were analyzed by LC ESI-MS/MS and the area under the curve (AUC) recorded. Phosphatidylcholines were normalized against PC (17∶0–14∶1) internal standard. Phosphatidylethanolamines were normalized with PE (12∶0–13∶0).The linearity was determined from 4 different concentrations. At each concentration, 3 replicates were analyzed and the mean value is reported.
(DOCX)

Figure S2 Total ion chromatogram (**A**) and extracted ion chromatogram (**B**) of 874.78 m/z from a NASH hepatic extract.
(DOCX)

Figure S3 **Immunohistochemical (IHC) staining patterns in normal human liver for various enzymes involved in lipid metabolism.** IHC images available at the Human Protein Atlas were interrogated for the zonal expression of 87 different enzymes mediating fatty acid oxidation and transport, triglyceride metabolism and phospholipid metabolism. Shown are representative IHC images for enzymes displaying the most pronounced zonation. **A–F**) Fatty acid oxidation and transport proteins such as CPT1B, CRAT, SLC25A20, ACADVL, DECR1 displayed strong perivenular (zone 3) to periportal (zone 1) expression patterns while ACACB (**F**) displayed the converse pattern (zone 1 to zone 3). **G–H**) Proteins involved in triglyceride metabolism such as DGAT2 and PLIN displayed zone 3 to zone 1 distributions. I–P) Several proteins facilitating phospholipid metabolism, specifically CHKA, PCYT1B, PEMT, PCYT2, GPAT2, PLA2G15, PLA2G4F and PLA2B1 all displayed strong zonation. In each instance, except for PCYT2, the strongest staining was in zone 3.
(DOCX)

Figure S4 **Portal tracts imaged by MALDI-IMS display robust levels of PC 32∶0.** Photomicrographs of H&E stained sections from each specimen (**A, C, E**) and corresponding MALDI IMS images of selected liver specimen obtained from subjects with normal (**A–D**) and NASH (**E–F**) histologies. (**B, D, F**) MALDI image of *m/z* 772.52 PC 32∶0 [M+K]^+^. Ion intensity color scale for all ion images is shown at the top of the figure. Scale bar = 500 µm.
(DOCX)

Table S1 Fragmentation and LC retention time information.
(DOCX)

Table S2 Lipids Identified and Quantified in Hepatic Extracts by LC ESI-MS/MS.
(DOCX)

Table S3 List of proteins identified in normal human liver and their expression profile in the Human Protein Atlas.
(DOCX)

Methods S1 (DOCX)
