# Supplementary material for: Differential Intrahepatic Phospholipid Zonation in Simple Steatosis and Nonalcoholic Steatohepatitis
Source: PLoS One. 2013 Feb 25;8(2):e57165. doi: 10.1371/journal.pone.0057165 (PMC3581520; doi:10.1371/journal.pone.0057165)
Supplement: Figure S1 — Calibration curves for lipid standards were determined at the concentration range indicated. Increasing amounts of A) 1-heptadecanoyl-2-(9Z-tetradecenoyl)-sn-glycero-3-phosphocholine (PC 17∶0–14∶1) and B) 1-dodecanoyl-2-tridecanoyl-sn-glycero-3-phosphoethanolamine (PE 12∶0–13∶0) were analyzed by LC ESI-MS/MS and the area under the curve (AUC) recorded. Phosphatidylcholines were normalized against PC (17∶0–14∶1) internal standard. Phosphatidylethanolamines were normalized with PE (12∶0–13∶0).The linearity was determined from 4 different concentrations. At each concentration, 3 replicates were analyzed and the mean value is reported. (DOCX) [file pone.0057165.s001.docx]

**A**

**B**

**Figure S1.**
