# Supplementary figures and images for: Differential Intrahepatic Phospholipid Zonation in Simple Steatosis and Nonalcoholic Steatohepatitis
Source: PLoS One. 2013 Feb 25;8(2):e57165. doi: 10.1371/journal.pone.0057165 (PMC3581520; doi:10.1371/journal.pone.0057165)

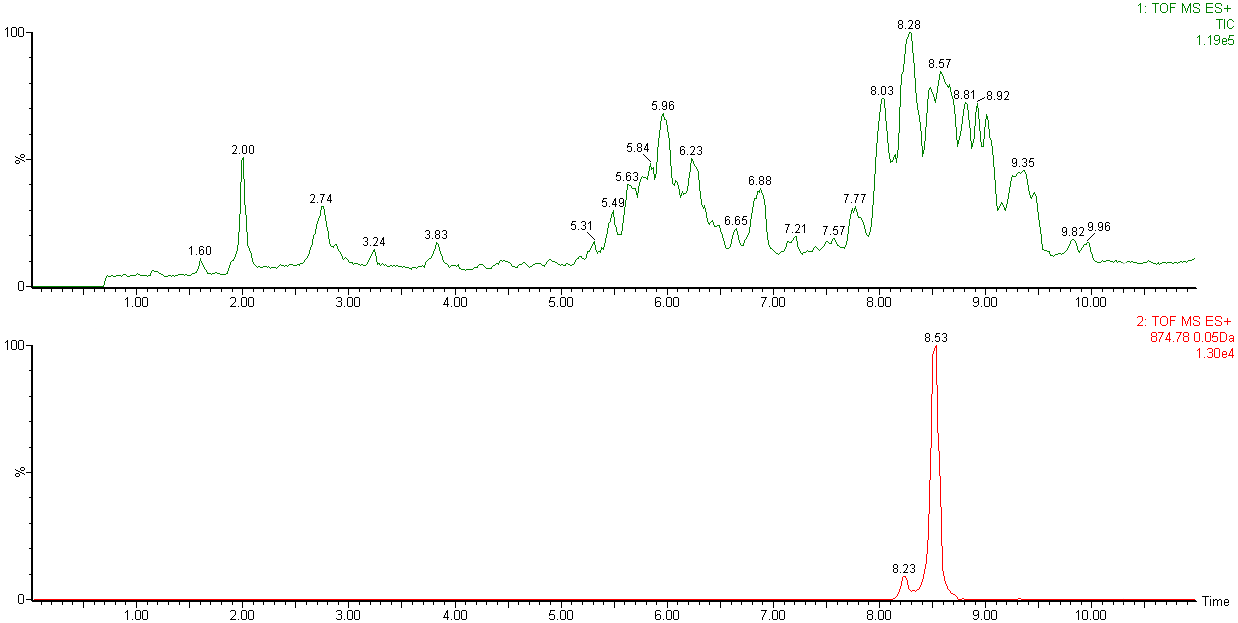


**Relative intensity**

**Relative intensity**

**Time**

**Figure S2.**

Supplement: Figure S2 — Total ion chromatogram (A) and extracted ion chromatogram (B) of 874.78 m/z from a NASH hepatic extract. (DOCX) [file pone.0057165.s002.docx]

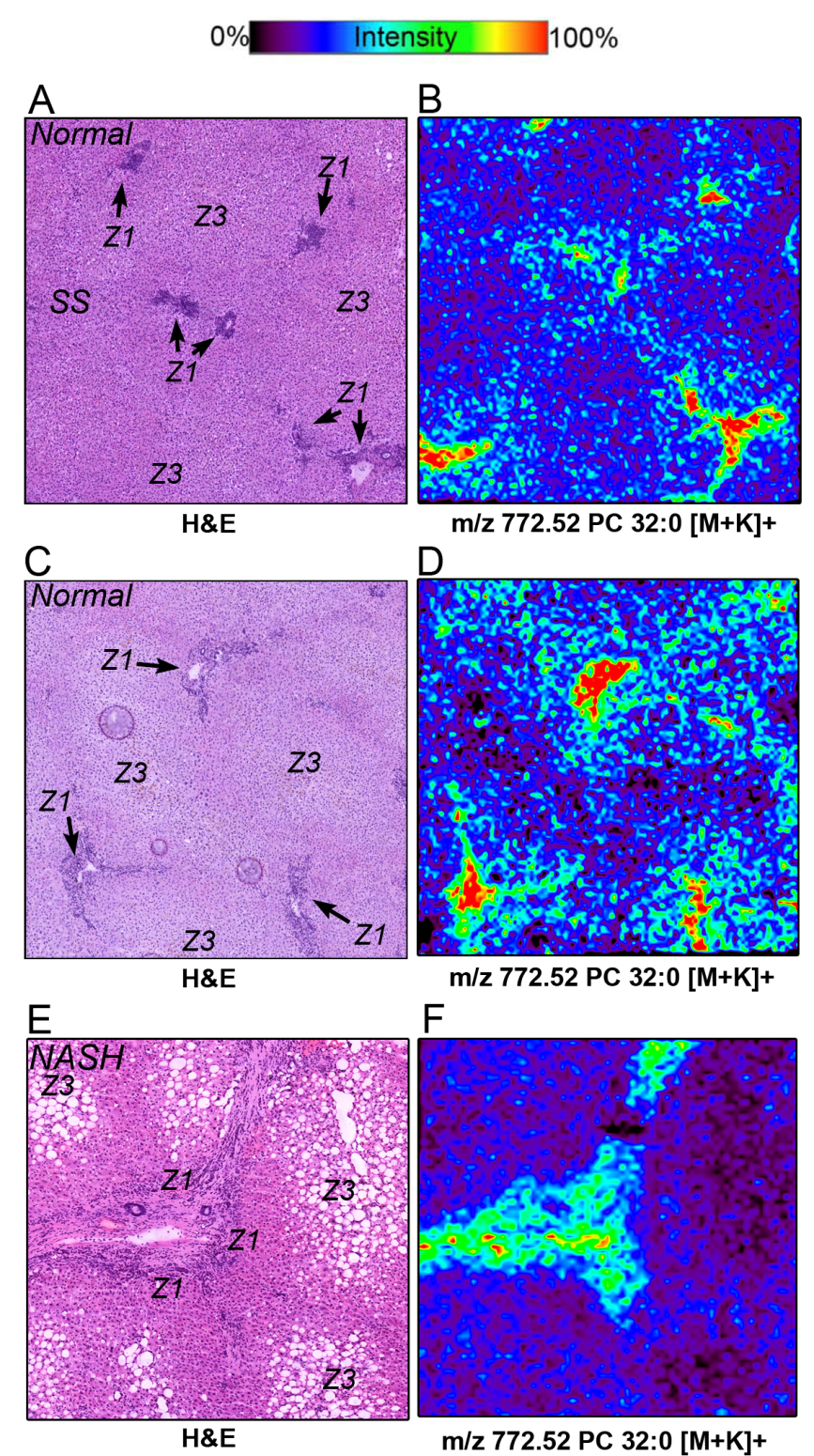


**Figure S4.**

Supplement: Figure S4 — Portal tracts imaged by MALDI-IMS display robust levels of PC 32∶0. Photomicrographs of H&E stained sections from each specimen (A, C, E) and corresponding MALDI IMS images of selected liver specimen obtained from subjects with normal (A–D) and NASH (E–F) histologies. (B, D, F) MALDI image of m/z 772.52 PC 32∶0 [M+K]+. Ion intensity color scale for all ion images is shown at the top of the figure. Scale bar = 500 µm. (DOCX) [file pone.0057165.s004.docx]
