# Supplementary material for: Differential Intrahepatic Phospholipid Zonation in Simple Steatosis and Nonalcoholic Steatohepatitis
Source: PLoS One. 2013 Feb 25;8(2):e57165. doi: 10.1371/journal.pone.0057165 (PMC3581520; doi:10.1371/journal.pone.0057165)
Supplement: Figure S3 — Immunohistochemical (IHC) staining patterns in normal human liver for various enzymes involved in lipid metabolism. IHC images available at the Human Protein Atlas were interrogated for the zonal expression of 87 different enzymes mediating fatty acid oxidation and transport, triglyceride metabolism and phospholipid metabolism. Shown are representative IHC images for enzymes displaying the most pronounced zonation. A–F) Fatty acid oxidation and transport proteins such as CPT1B, CRAT, SLC25A20, ACADVL, DECR1 displayed strong perivenular (zone 3) to periportal (zone 1) expression patterns while ACACB (F) displayed the converse pattern (zone 1 to zone 3). G–H) Proteins involved in triglyceride metabolism such as DGAT2 and PLIN displayed zone 3 to zone 1 distributions. I–P) Several proteins facilitating phospholipid metabolism, specifically CHKA, PCYT1B, PEMT, PCYT2, GPAT2, PLA2G15, PLA2G4F and PLA2B1 all displayed strong zonation. In each instance, except for PCYT2, the strongest staining was in zone 3. (DOCX) [file pone.0057165.s003.docx]

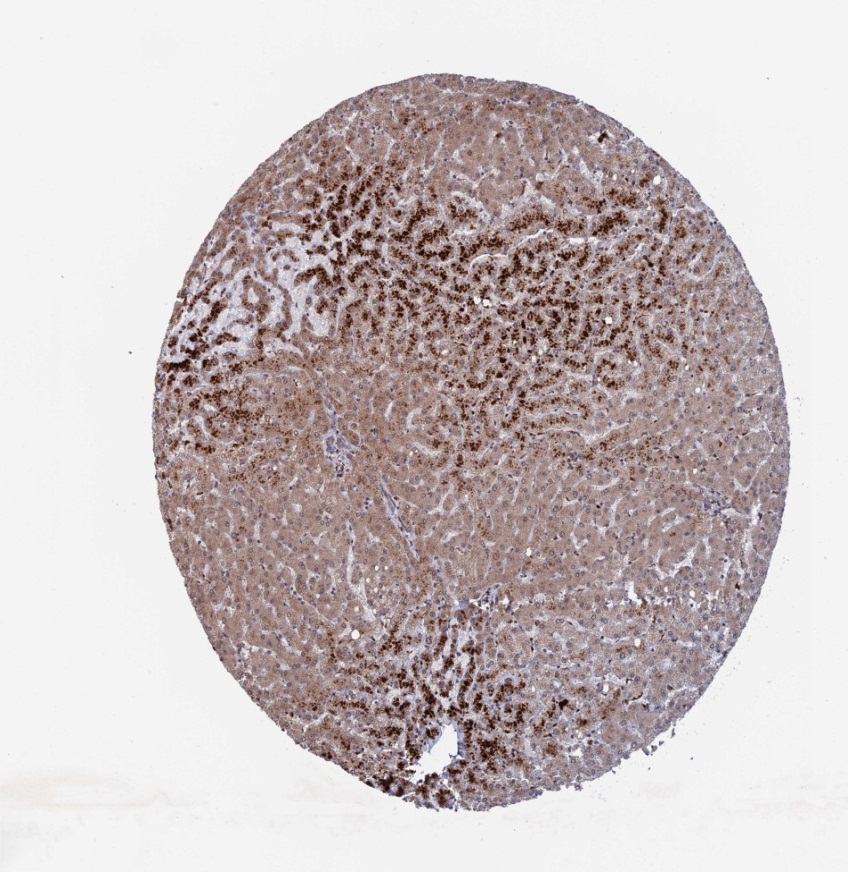


**N**

Phospholipase A2 G15


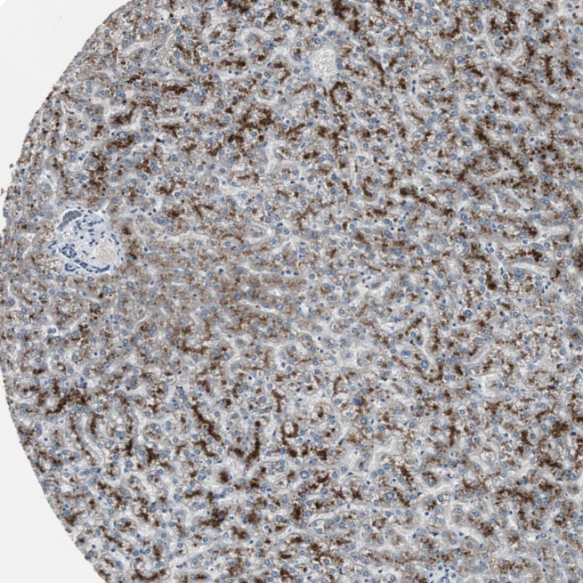


**J**

PCYT1B


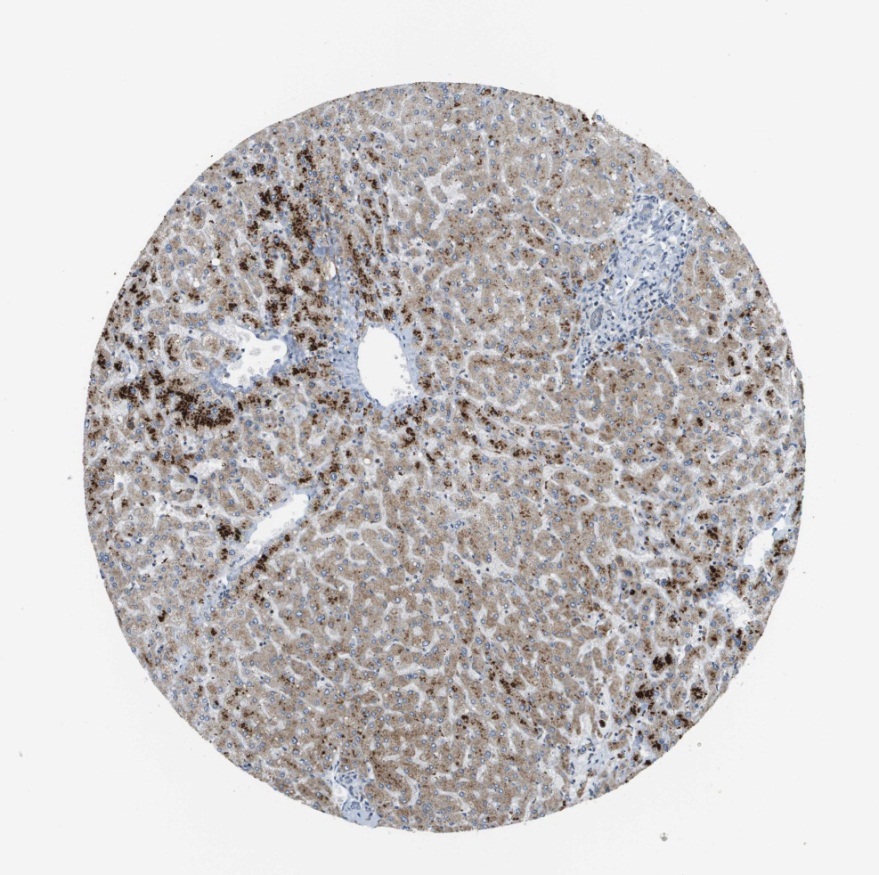


**C**

SLC25A20


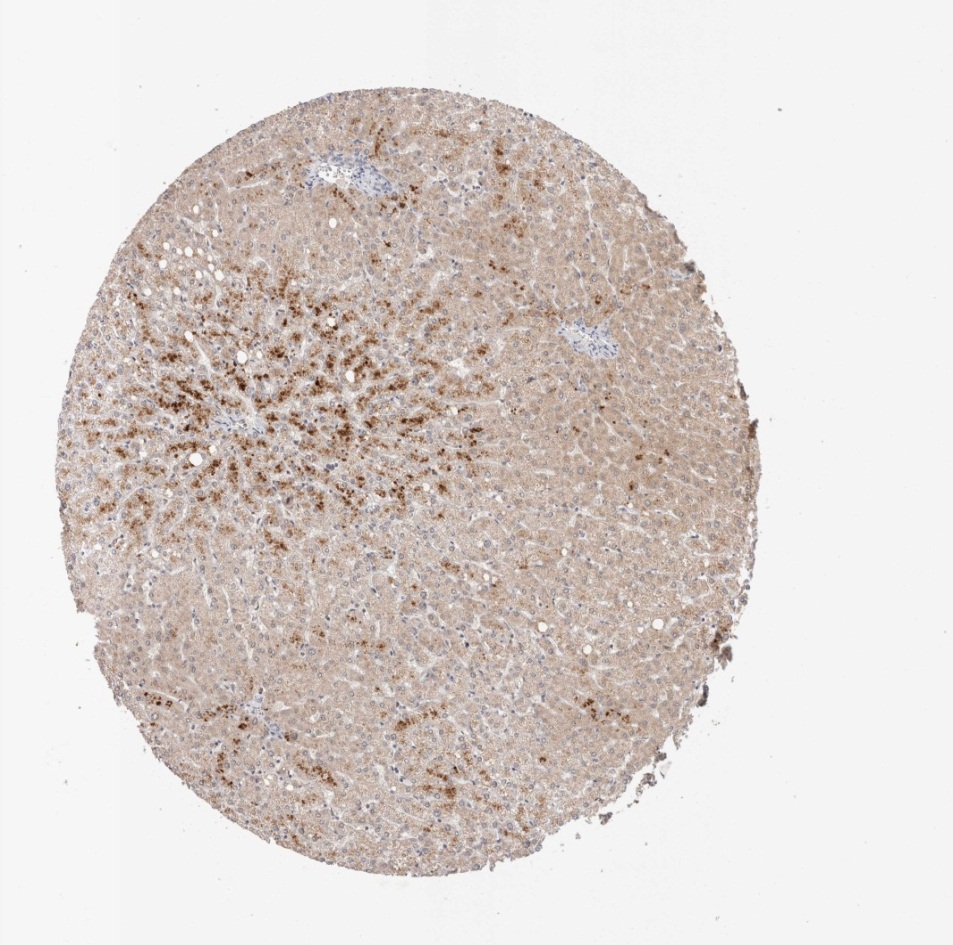


**B**

CRAT


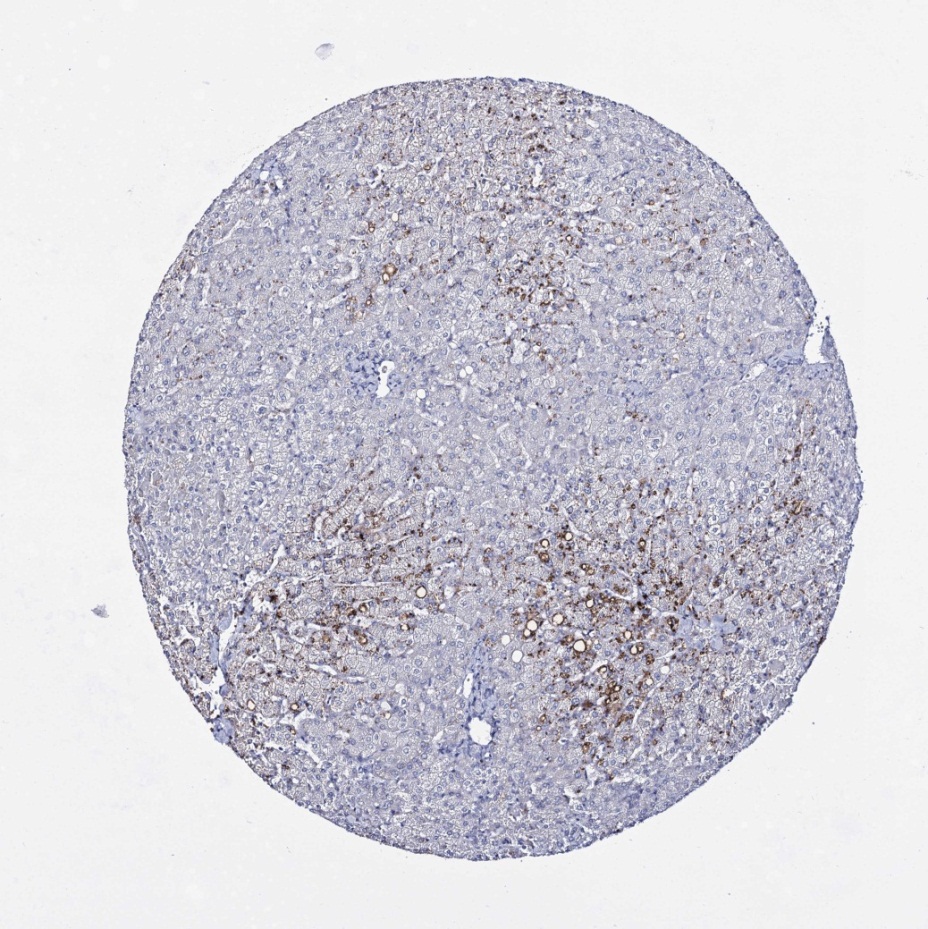


**H**

PLIN


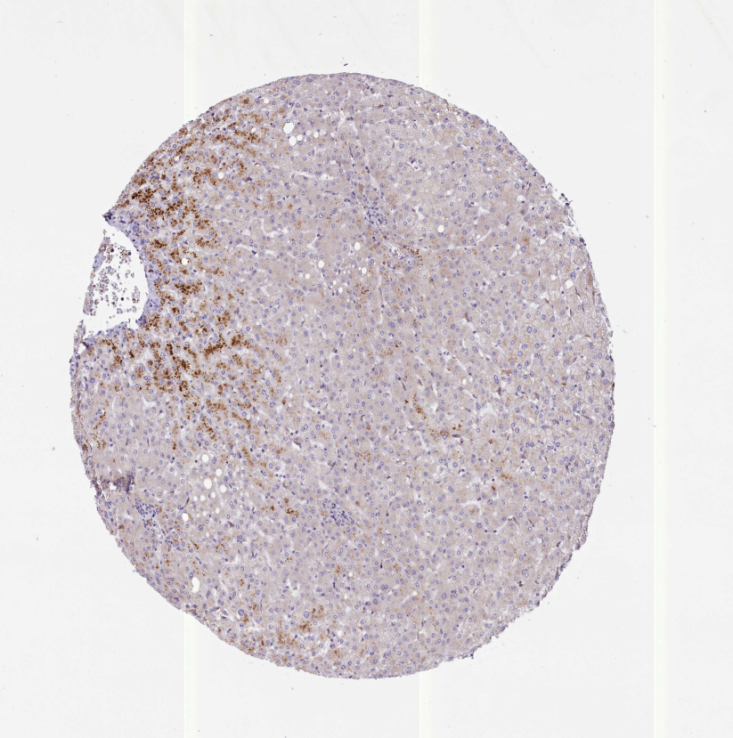


**K**

PEMT


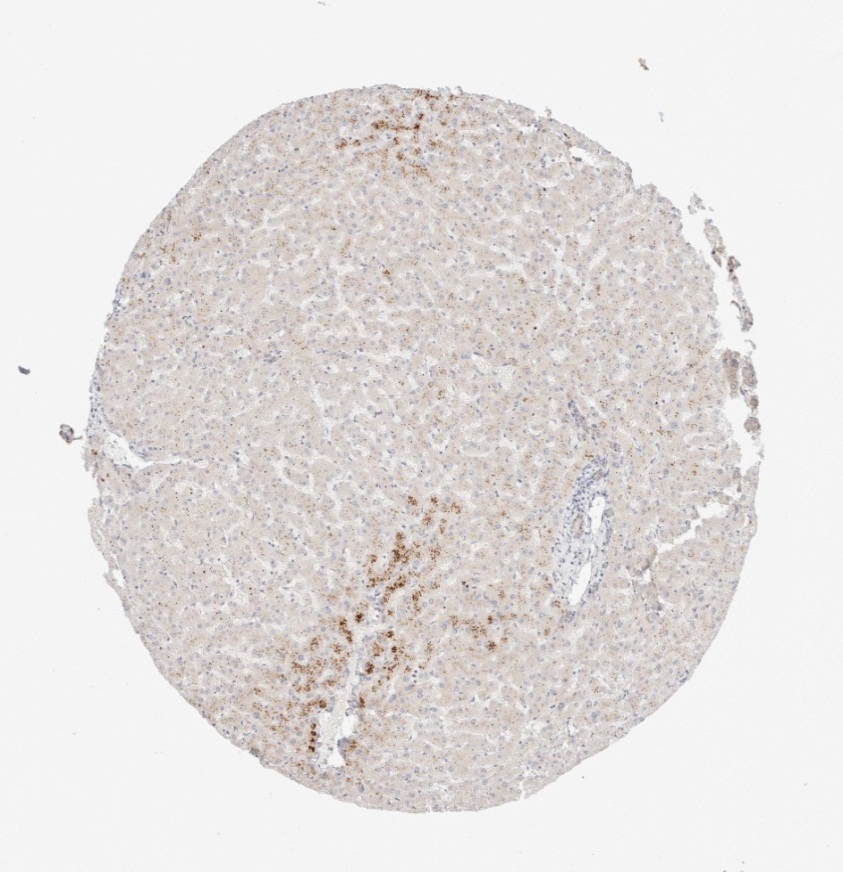


**I**

CHKA


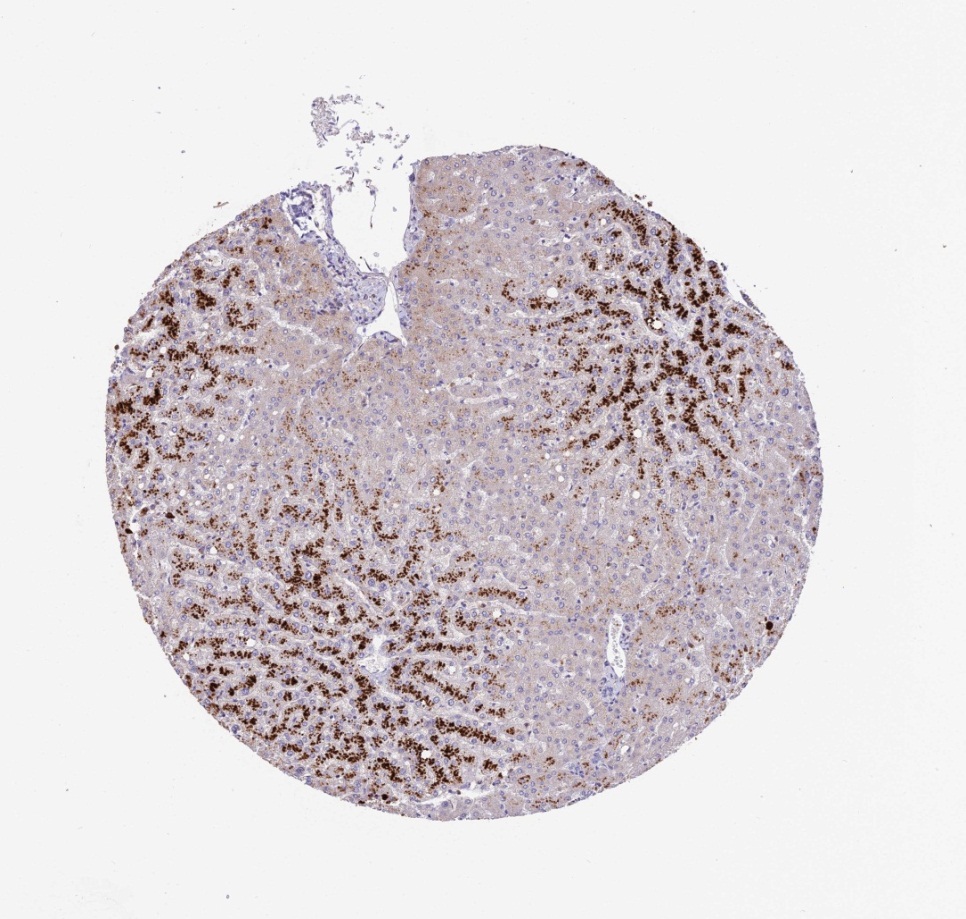


**O**

Phospholipase A2 G4F


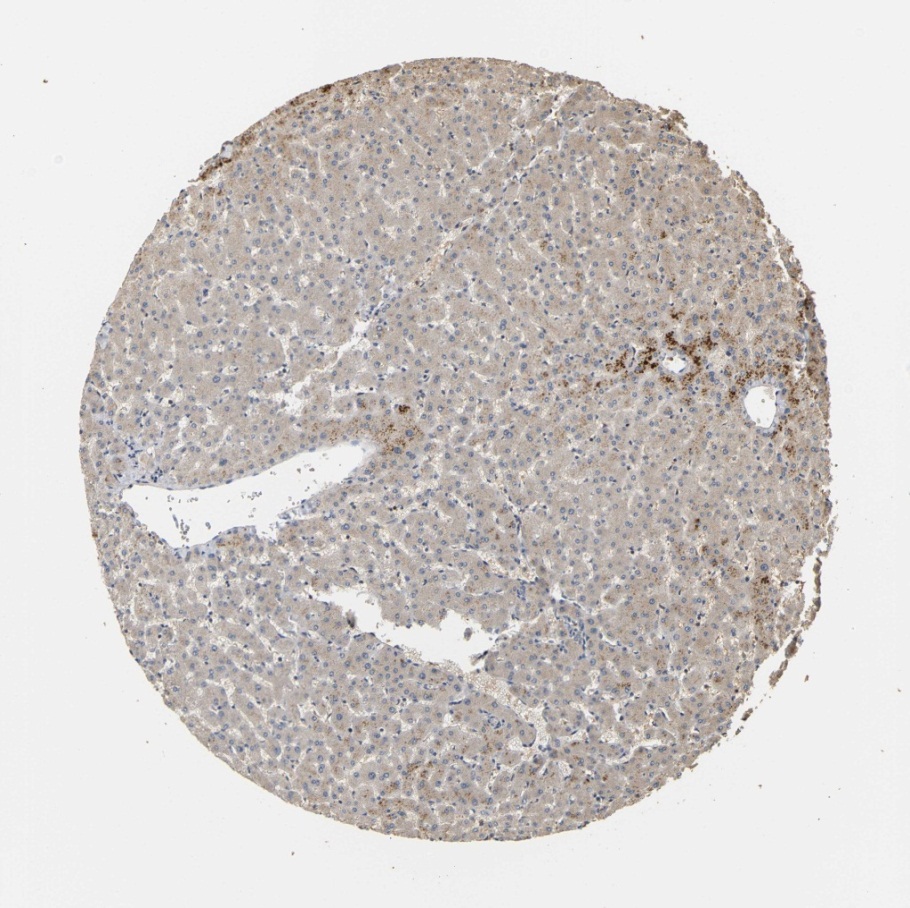


**P**

Phospholipase B1


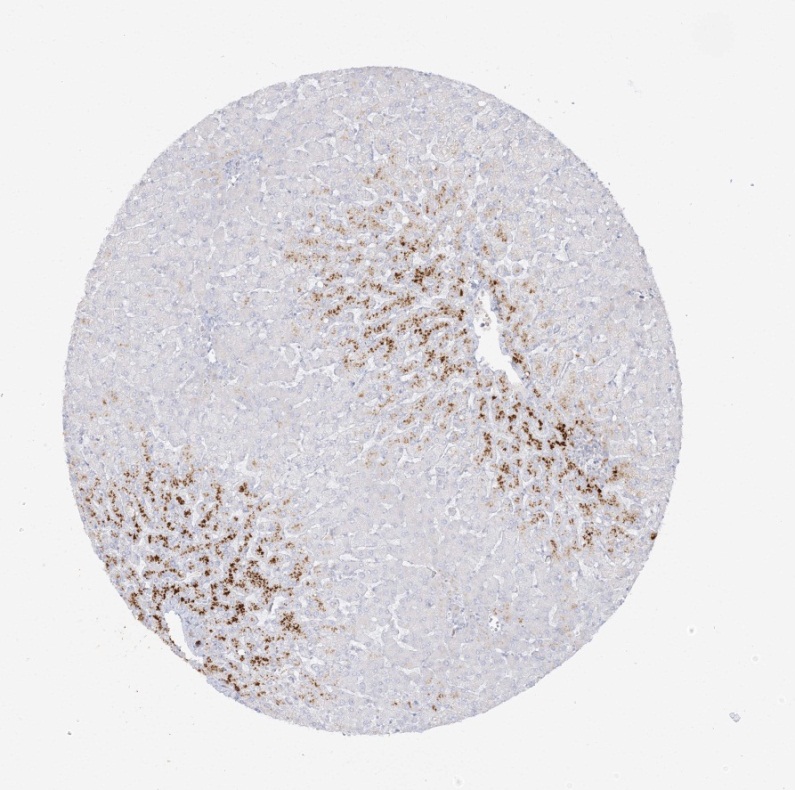


**M**

GPAT2


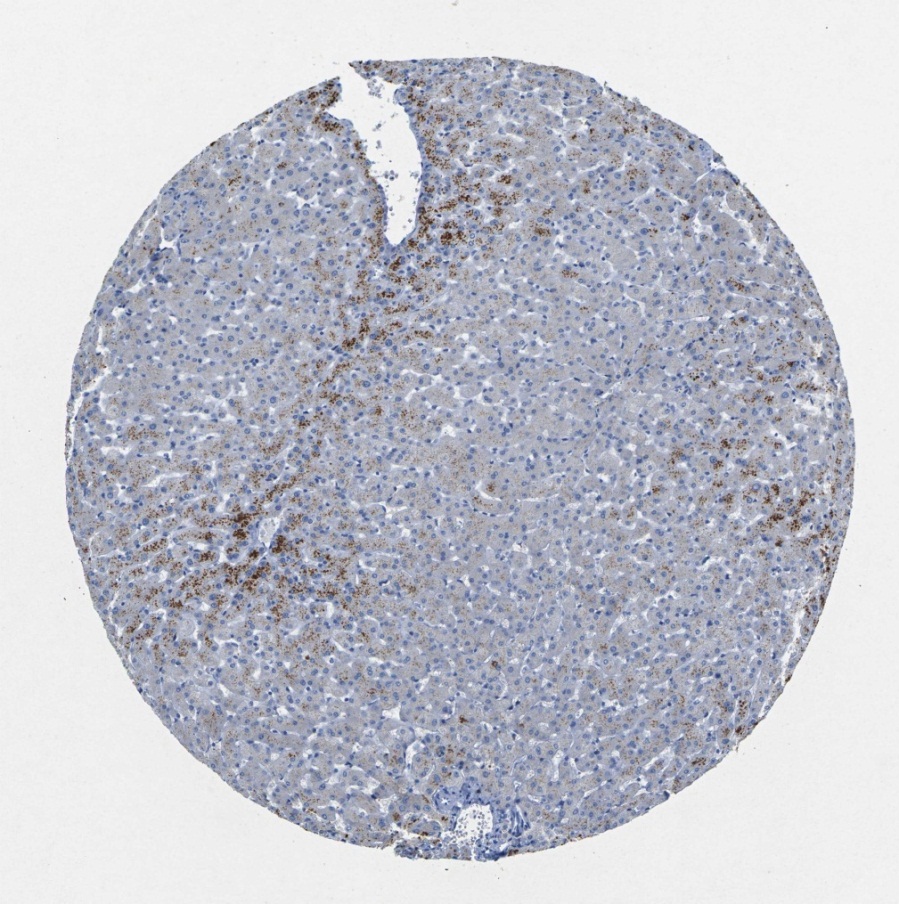


**G**

DGAT2


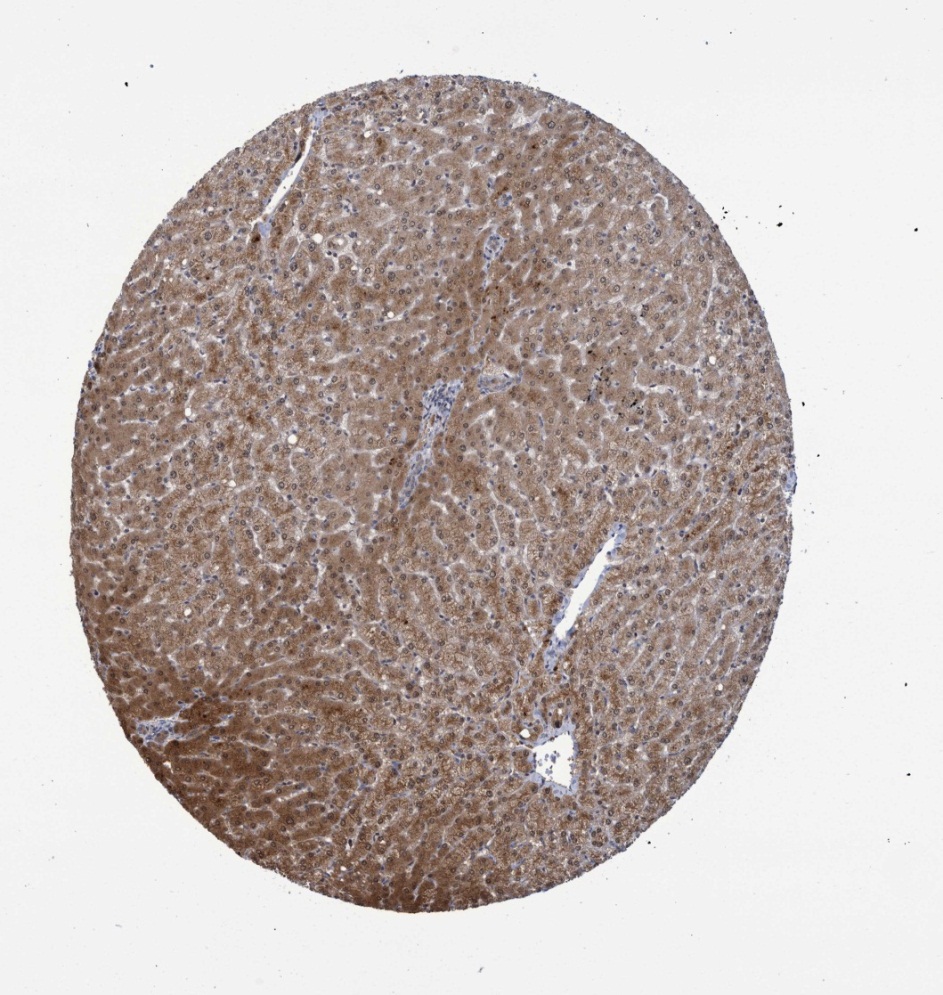


**L**

PCYT2


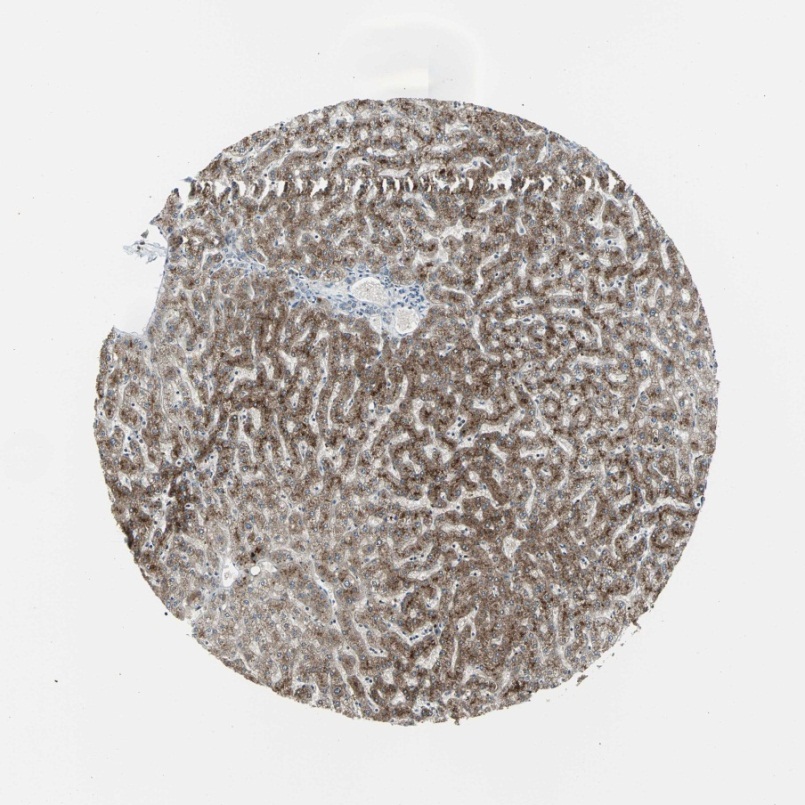


**F**

ACACB


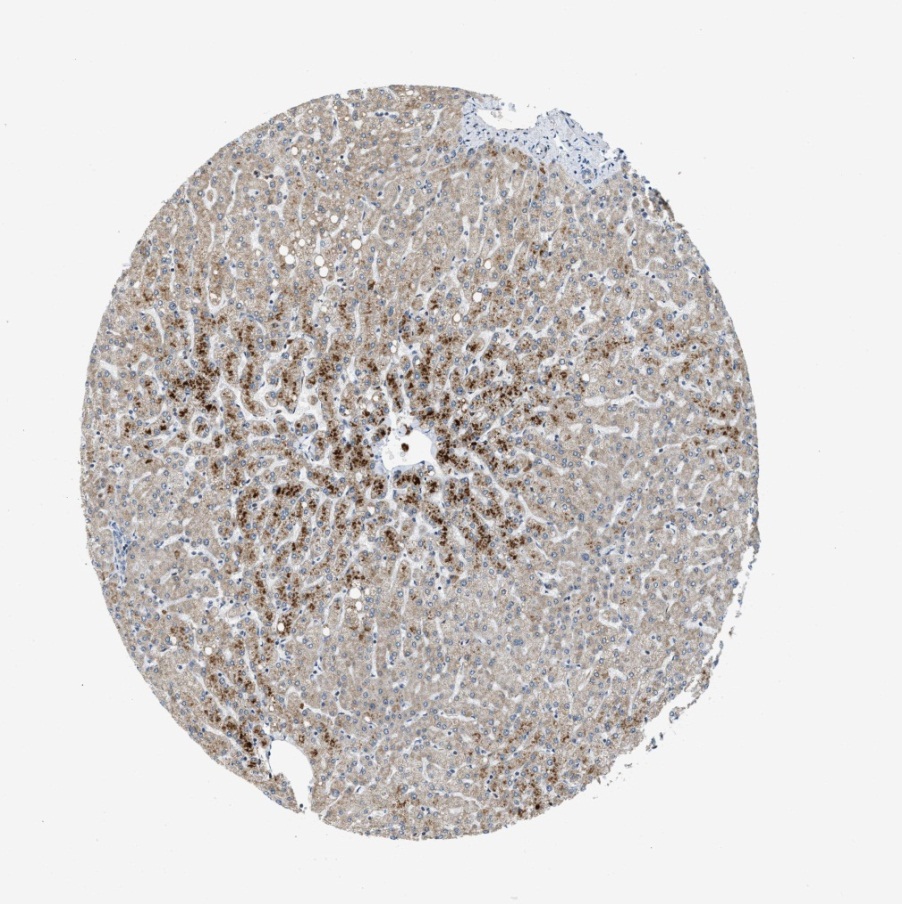


**D**

ACADVL


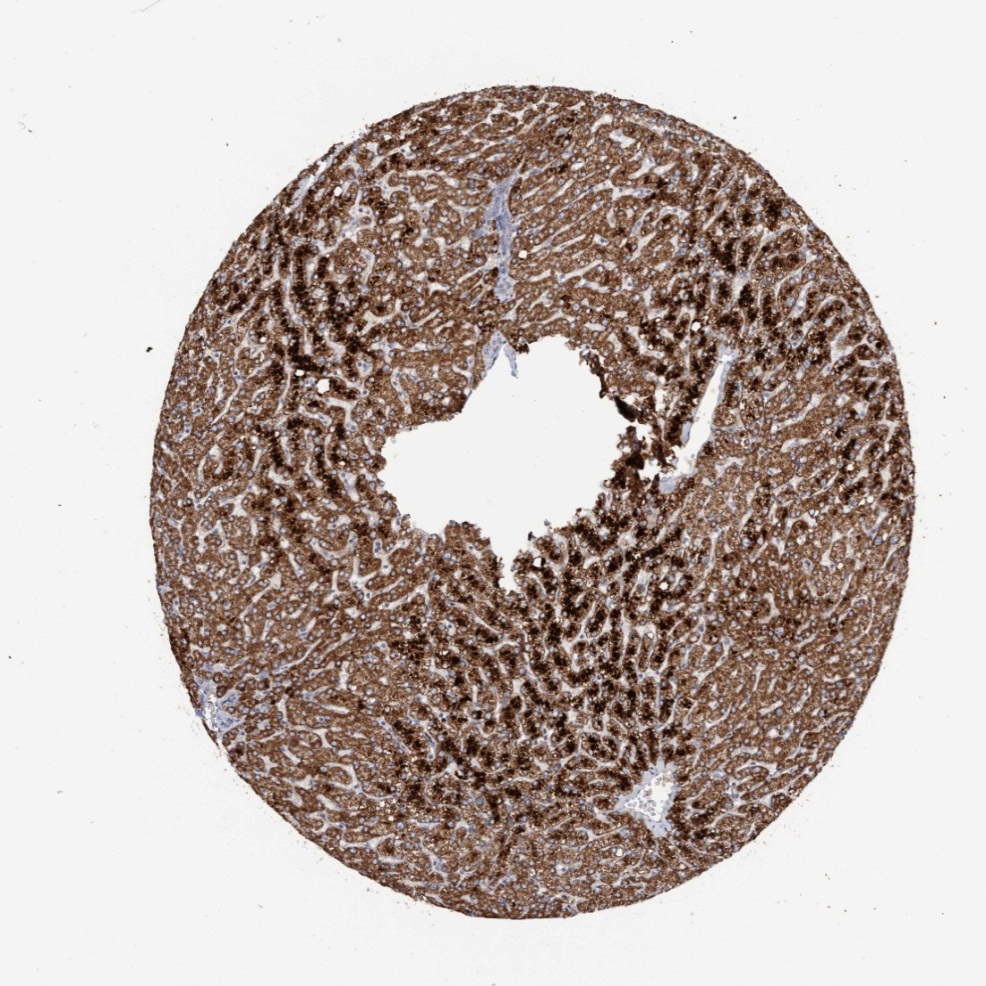


**E**

DECR1


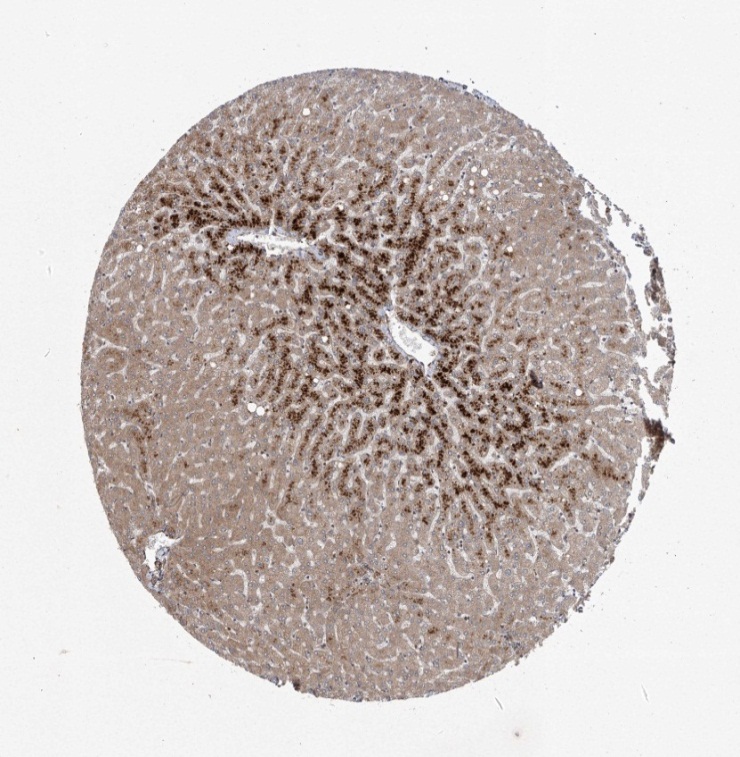


**A**

CPT1B

**Figure S3.**
