# Supplementary material for: Differential Intrahepatic Phospholipid Zonation in Simple Steatosis and Nonalcoholic Steatohepatitis
Source: PLoS One. 2013 Feb 25;8(2):e57165. doi: 10.1371/journal.pone.0057165 (PMC3581520; doi:10.1371/journal.pone.0057165)
Supplement: Table S1 — Fragmentation and LC retention time information. (DOCX) [file pone.0057165.s005.docx]

| **Table S1. Fragmentation and LC retention time information** | | | | | |
| --- | --- | --- | --- | --- | --- |
| **Compound** | **LipidMaps ID** | **Monoisotopic mass (m/z)** | **Detected*** | **MS/MS ions** | **Retention time (min)** |
| PC (17:0-14:1) | LM-1004 | 717.53 | **[M+H]^+^** | 184.07 | 5.55 |
| PE (12:0-13:0) | LM-1100 | 593.40 | **[M-140] ^+^** | 186.22 | 4.44 |
| PCs |  |  | **[M+H]^+^** | 184.07 |  |
| PEs |  |  | **[M-140] ^+^**, [M+H]^+^ | 186.22 |  |

**Most intense ions marked in bold*
