# Supplementary material for: Differential Intrahepatic Phospholipid Zonation in Simple Steatosis and Nonalcoholic Steatohepatitis
Source: PLoS One. 2013 Feb 25;8(2):e57165. doi: 10.1371/journal.pone.0057165 (PMC3581520; doi:10.1371/journal.pone.0057165)
Supplement: Table S2 — Lipids Identified and Quantified in Hepatic Extracts by LC ESI-MS/MS. (DOCX) [file pone.0057165.s006.docx]

| **Table S2. Lipids Identified and Quantified in Hepatic Extracts by LC ESI-MS/MS** | | | | | | |  |  |
| --- | --- | --- | --- | --- | --- | --- | --- | --- |
|  |  |  |  | **Ret. Time** | **Observed** | **Theoretical** |  |  |
| **Ion** | **Class** | **Species** | **Formula** | **(min)** | ***m/z*** | ***m/z*** | **ppm** | **Significant** |
| [M+H]^+^ | PA | 32:1 | C35H68O8P | 6.89 | 647.4615 | 647.4646 | 4.8 | yes |
| [M+H]^+^ | PC | 32:0 | C40H81NO8P | 6.26 | 734.5639 | 734.5694 | 7.5 | no |
| [M+H]^+^ | PC | 32:1 | C40H79NO8P | 5.75 | 732.5483 | 732.5537 | 7.4 | no |
| [M+H]^+^ | PC | 34:1 | C42H83NO8P | 6.24 | 760.5822 | 760.5850 | 3.7 | no |
| [M+H]^+^ | PC | 34:2 | C42H81NO8P | 5.82 | 758.5641 | 758.5694 | 7.0 | yes |
| [M+H]^+^ | PC | 34:3 | C42H79NO8P | 5.36 | 756.5476 | 756.5537 | 8.1 | yes |
| [M+H]^+^ | PC | 34:6 | C42H73NO8P | 6.01 | 750.5059 | 750.5068 | 1.2 | yes |
| [M+H]^+^ | PC | 35:2 | C43H83NO8P | 6.06 | 772.5804 | 772.5850 | 6.0 | no |
| [M+H]^+^ | PC | 36:1 | C44H87NO8P | 6.70 | 788.6102 | 788.6163 | 7.7 | yes |
| [M+H]^+^ | PC | 36:2 | C44H84NO7P | 6.32 | 786.6023 | 786.6007 | -2.0 | yes |
| [M+H]^+^ | PC | 36:3 | C44H83NO8P | 5.93 | 784.5852 | 784.5850 | -0.3 | no |
| [M+H]^+^ | PC | 36:4 | C44H81NO8P | 5.69 | 782.5666 | 782.5694 | 3.6 | yes |
| [M+H]^+^ | PC | 36:5 | C44H79NO8P | 5.78 | 780.5524 | 780.5537 | 1.7 | no |
| [M+H]^+^ | PC | 38:3 | C46H86NO7P | 6.38 | 812.6118 | 812.6163 | 5.5 | no |
| [M+H]^+^ | PC | 38:4 | C46H85NO8P | 6.17 | 810.5963 | 810.6007 | 5.4 | no |
| [M+H]^+^ | PC | 38:5 | C46H83NO8P | 5.66 | 808.5808 | 808.5850 | 5.2 | no |
| [M+H]^+^ | PC | 38:6 | C46H81NO8P | 5.50 | 806.5670 | 806.5694 | 3.0 | yes |
| [M+H]^+^ | PC | 38:7 | C46H79NO8P | 5.67 | 804.5496 | 804.5537 | 5.1 | no |
| [M+H]^+^ | PC | 40:0 | C48H97NO8P | 8.28 | 846.6954 | 846.6946 | -0.9 | yes |
| [M+H]^+^ | PC | 40:6 | C48H85NO8P | 5.97 | 834.5945 | 834.6007 | 7.4 | yes |
| [M+H]^+^ | PE | 28:1 | C33H65NO8P | 2.65 | 634.4500 | 634.4442 | -9.1 | yes |
| [M+H]^+^ | PE | 34:2 | C39H75NO8P | 5.99 | 716.5155 | 716.5224 | 9.6 | yes |
| [M+H]^+^ | PE | 34:6 | C39H67NO8P | 5.98 | 708.4663 | 708.4598 | -9.2 | no |
| [M+H]^+^ | PE | 36:2 | C41H79NO8P | 6.45 | 744.5487 | 744.5537 | 6.7 | no |
| [M+H]^+^ | PE | 36:4 | C41H75NO8P | 5.82 | 740.5161 | 740.5224 | 8.5 | no |
| [M+H]^+^ | PE | 38:4 | C43H79NO8P | 6.33 | 768.5488 | 768.5537 | 6.4 | no |
| [M+H]+ | PE | 38:5 | C43H77NO8P | 2.60 | 766.5401 | 766.5381 | -2.6 | yes |
| [M+H]^+^ | PE | 38:6 | C43H75NO8P | 5.61 | 764.5155 | 764.5224 | 9.0 | yes |
| [M+H]^+^ | PE | 40:6 | C45H79NO8P | 6.12 | 792.5480 | 792.5537 | 7.2 | yes |
| [M+H]^+^ | PG | 32:1 | C47H77O5 | 5.97 | 721.4948 | 721.5014 | 9.1 | no |
| Abbreviations: PC - phosphatidylcholine, PE - phosphatidylethanolamine; *m/z* - mass/charge. | | | | | | |  |  |
| Significance = P < 0.05 by Student's t-test with Bonferroni correction for multiple comparisons. | | | | | | |  |  |
