# Supplementary material for: Differential Intrahepatic Phospholipid Zonation in Simple Steatosis and Nonalcoholic Steatohepatitis
Source: PLoS One. 2013 Feb 25;8(2):e57165. doi: 10.1371/journal.pone.0057165 (PMC3581520; doi:10.1371/journal.pone.0057165)
Supplement: Table S3 — List of proteins identified in normal human liver and their expression profile in the Human Protein Atlas. (DOCX) [file pone.0057165.s007.docx]

**Table S3. List of Proteins Identified in Normal Human Liver and Their Expression Profile in the Human Protein Atlas**

| **Pathway** | **Acc. No.** | **Protein Name** | **Gene Symbol** | **HPA antibody** | **Intensity** | **Zonation in normal tissue** |
| --- | --- | --- | --- | --- | --- | --- |
| **FA oxidation & transport** | P16671 | CD36 molecule (thrombospondin receptor) | CD36 | HPA002018  CAB025866 | negative  negative | none |
|  | P50416 | carnitine palmitoyltransferase 1A (liver) | CPT1A | HPA008835 | moderate | none |
|  | Q92523 | carnitine palmitoyltransferase 1B (muscle) | CPT1B | HPA029583 | weak | **pronounced** |
|  | P23786 | carnitine palmitoyltransferase 2 | CPT2 | HPA028201  HPA028202  HPA028214 | strong  moderate  negative | none  none  none |
|  | P33121 | acyl-CoA synthetase long-chain family member 1 | ACSL1 | HPA011316  HPA011964 | strong  strong | none  none |
|  | O95573 | acyl-CoA synthetase long-chain family member 3 | ACSL3 | HPA011315 | strong | none |
|  | O60488 | acyl-CoA synthetase long-chain family member 4 | ACSL4 | HPA005552 | moderate | none |
|  | Q9ULC5 | acyl-CoA synthetase long-chain family member 5 | ACSL5 | - | - | - |
|  | Q9UKU0 | acyl-CoA synthetase long-chain family member 6 | ACSL6 | HPA040470 | moderate | none |
|  | P43155 | carnitine acyltransferase | CRAT | HPA019230  HPA020260  HPA022815 | weak  weak  moderate | none  none  **pronounced** |
|  | O43772 | carnitine/acylcarnitine transporter, member 20 | SLC25A20 | HPA016862  HPA029683 | moderate  strong | **pronounced**  none |
|  | P49748 | acyl-CoA dehydrogenase, very long chain | ACADVL | HPA019006  HPA020595 | moderate  moderate | **pronounced**  **pronounced** |
|  | P11310 | acyl-CoA dehydrogenase, C-4 to C-12 straight chain | ACADM | HPA006198  HPA026542 | strong  strong | none  none |
|  | P16219 | acyl-CoA dehydrogenase, C-2 to C-3 short chain | ACADS | HPA004799  CAB019284  HPA022271 | moderate  strong  strong | none  none  none |
|  | P45954 | acyl-CoA dehydrogenase, C-2 to C-3 short/branched chain | ACADSB | - | - | - |
|  | P40939 | hydroxyacyl-CoA dehydrogenase, α subunit | HADHA | HPA015536 | moderate | none |
|  | P55084 | hydroxyacyl-CoA dehydrogenase, β subunit | HADHB | HPA037539 | strong | none |
|  | Q16698 | 2,4 dienoyl-CoA reductase isoform 1 | DECR1 | HPA023160 HPA023162 HPA023238 | strong  strong  strong | **pronounced**  none  none |
|  | P42146 | enoyl-CoA delta isomerase 1 | ECI1 | HPA041746  HPA043227 | strong  strong | none  none |
|  | O75521 | enoyl-CoA delta isomerase 2 | ECI2 | HPA022130 | strong |  |
|  | Q13011 | 2-enoyl-CoA hydratase isoform 1 | ECH1 | HPA002907  HPA005835 | strong  strong | none  none |
|  | P30084 | 2-enoyl-CoA hydratase isoform | ECHS1 | CAB003783  HPA021995  HPA022476 | strong  strong  strong | none  none  none |
|  | Q16836 | 3-hydroxyacyl-CoA dehydrogenase | HADH | HPA039588 | strong | none |
|  | Q99714 | hydroxysteroid (17-beta) dehydrogenase 10 | HSD17B10 | HPA001432 | moderate | **mild** |
|  | P42765 | acetyl-CoA acyltransferase 2 | ACAA2 | HPA042303 | strong | **mild** |
|  | O00767 | stearoyl-coA desaturase isoform 1 | SCD1 | HPA012107 | moderate | none |
|  | Q86SK9 | stearoyl-coA desaturase isoform 5 | SCD5 | HPA042380 | strong | none |
| **Triglyceride metabolism** | P21695 | glycerol-3-phosphate dehydrogenase 1 (soluble) | GPD1 | HPA044620 | strong | none |
|  | P43304 | glycerol-3-phosphate dehydrogenase 2 | GPD2 | HPA008012 | weak | none |
|  | Q9HCL2 | glycerol-3-phosphate acyltransferase, mitochondrial | GPAM | HPA046339 | moderate | none |
|  | O15120 | 1-acylglycerol-3-phosphate O-acyltransferase 2 (lysophosphatidic acid acyltransferase, β | AGPAT2 | HPA019544 | weak | none |
|  | Q53H12 | acylglycerol kinase | AGK | HPA020959 | negative | none |
|  | P23743 | diacylglycerol kinase alpha | DGKA | HPA041645 | negative | none |
|  | Q9Y6T7 | diacylglycerol kinase beta | DGKB | - | - | - |
|  | P49619 | diacylglycerol kinase gamma | DGKG | HPA036577 | negative | none |
|  | P52429 | diacylglycerol kinase epsilon | DGKE | HPA017167 | negative | none |
|  | P52824 | diacylglycerol kinase theta | DGKQ | HPA026797 | negative | none |
|  | Q13574 | diacylglycerol kinase zeta | DGKZ | HPA051336 | negative | none |
|  | O75907 | diacylglycerol O-acyltransferase 1 | DGAT1 | CAB032853 | moderate | none |
|  | Q96PD7 | diacylglycerol O-acyltransferase 2 | DGAT2 | HPA013351 | moderate | **pronounced** |
|  | Q96PD6 | monoacylglycerol O-acetyltransferase 1 | MOGAT1 | HPA049944 | negative | none |
|  | Q3SYC2 | monoacylglycerol O-acetyltransferase 2 | MOGAT2 | HPA028834 | weak | none |
|  | Q86VF5 | monoacylglycerol O-acetyltransferase 3 | MOGAT3 | HPA011940 | weak | **mild** |
|  | P49327 | fatty acid synthase | FASN | CAB005192  HPA006461  CAB015417 | weak  moderate  strong | none  none  **mild** |
|  | O00763 | acetyl-CoA carboxylase beta | ACACB | HPA006554 | strong | **pronounced** |
|  | Q13085 | acetyl-CoA carboxylase alpha | ACACA | CAB013715 | moderate | none |
|  | O95822 | malonyl-CoA decarboxylase | MLYCD | HPA031625 | moderate | **mild** |
|  | Q96AD5 | patatin-like phospholipase domain containing 2 | PNPLA2 | - | - | - |
|  | Q99685 | monoglyceride lipase | MGLL | HPA011993  HPA011994 | moderate  strong | none  none |
|  | O60427 | fatty acid desaturase 1 | FADS1 | HPA042705 | moderate | **mild** |
|  | O95864 | fatty acid desaturase 2 | FADS2 | HPA006741 | strong | none |
|  | Q9Y5Q0 | fatty acid desaturase 3 | FADS3 | HPA045224 | weak | none |
|  | O60240 | perilipin 1 | PLIN | HPA024299  CAB033821  CAB037333 | negative  negative  moderate | **pronounced**  **mild**  **pronounced** |
|  | Q99541 | perilipin 2 /adipophilin | PLIN2 | - | - | **-** |
|  | O60664 | perilipin 3/ TIP47 | PLIN3 | HPA006427 | weak | none |
|  | Q14693 | lipin 1 | LPIN1 | HPA038021 | moderate | **mild** |
|  | Q92539 | lipin 2 | LPIN2 | CAB015223HPA017857 | moderate  moderate | none  none |
|  | Q9BQK8 | lipin 3 | LPIN3 | - | - | - |
|  |  |  |  |  |  |  |
|  |  |  |  |  |  |  |
|  |  |  |  |  |  |  |
|  |  |  |  |  |  |  |
| **Phospholipid metabolism** | P35790 | choline kinase alpha | CHKA | HPA024153 | weak | **pronounced** |
|  | Q9Y259 | choline kinase beta | CHKB | HPA018797 | moderate | none |
|  | P495B5 | phosphocholine cytidylyltransferase α | PCYT1A | HPA035428 | moderate | none |
|  | Q9Y5K3 | phosphocholine cytidylyltransferase β | PCYT1B | HPA006367 | strong | **pronounced** |
|  | Q99447 | phosphocholine cytidylyltransferase 2 | PCYT2 | HPA023033  HPA023034 | moderate  moderate | **mild**  **mild** |
|  | Q9Y6K0 | choline ethanolamine phosphotransferase | CEPT1 | - | - | - |
|  | Q9UBM1 | phosphatidyl ethanolamine methyl transferase | PEMT | HPA042375 | weak | **pronounced** |
|  | Q8NCC3 | phospholipase A2 G15 | PLA2G15 | HPA041702  HPA041727 | weak  negative | **pronounced**  **pronounced** |
|  | Q68DD2 | phospholipase A2 G4F | PLA2G4F | HPA042713 | negative | **pronounced** |
|  | Q6P1J6 | phospholipase B1 | PLB1 | HPA014045 | moderate | **pronounced** |
|  | Q8IY17 | patatin-like phospholipase domain containing 6 protein | PNPLA6 | HPA007522 | moderate | **mild** |
|  | O75608 | lysophospholipase 1 | LYPLA1 | HPA050941 | moderate | none |
|  | O95372 | lysophospholipase 2 | LYPLA2 | - | - | - |
|  | Q6NUI2 | glycerol-3-phosphate acyltransferase | GPAT2 | HPA036841 | negative | **pronounced** |
|  | Q8NF37 | lysophosphatidylcholine acyltransferase 1 | LPCAT1 | HPA012501  HPA022268 | weak  weak | none  none |
|  | Q7L5N7 | lysophosphatidylcholine acyltransferase 2 | LPCAT2 | HPA007891 | weak | none |
|  | Q6P1A2 | lysophosphatidylcholine acyltransferase 3 | LPCAT3 | - | - | - |
|  | Q643R3 | lysophosphatidylcholine acyltransferase 4 | LPCAT4 | HPA030719 | moderate | **mild** |
|  | O14494 | phosphatidic acid phosphatase type 2A | PPAP2A | CAB033331 | weak | none |
|  | O14495 | phosphatidic acid phosphatase type 2B | PPAP2B | HPA028892 | negative | none |
|  | O14496 | phosphatidic acid phosphatase type 2C | PPAP2C | - | - | - |
|  | Q5VZY2 | phosphatidic acid phosphatase type 2 domain 1A | PPAPDC1A | HPA045188 | negative | none |
|  | Q8NEB5 | phosphatidic acid phosphatase type 2 domain 1B | PPAPDC1B | - | - | - |
|  | Q8IY26 | phosphatidic acid phosphatase type 2 domain 2 | PPAPDC2 | HPA018096 | weak | none |
|  | Q8NBP4 | phosphatidic acid phosphatase type 2 domain 3 | PPAPDC3 | - | - | - |
|  | Q96GM2 | lipid phosphate phosphatase-related protein type 2 | LPPR2 | HPA048973 | strong | none |
|  | Q6T4P5 | lipid phosphate phosphatase-related protein type 3 | LPPR3 | - | - | - |
|  | Q7Z2D5 | lipid phosphate phosphatase-related protein type 4 | LPPR4 | HPA008798 | weak | none |
