# Supplementary material for: Differential Intrahepatic Phospholipid Zonation in Simple Steatosis and Nonalcoholic Steatohepatitis
Source: PLoS One. 2013 Feb 25;8(2):e57165. doi: 10.1371/journal.pone.0057165 (PMC3581520; doi:10.1371/journal.pone.0057165)
Supplement: Methods S1 — (DOCX) [file pone.0057165.s008.docx]

**Supplemental Methods**

***Lipid Extraction and HPLC ESI-MS Data Acquisition***

The lipid standards PC 17:0-14:1 and PE 12:0-13:0 (250 nm of each lipid final) were added to 100 mg portions of freeze-dried liver tissue with 1 ml of Folch reagent (2:1, v/v chloroform/methanol) in 2 ml tubes containing lysing Matrix D ceramic beads. Liver samples were homogenized in a TeSe PRECESS 24 homogenizer (Bio-Rad) at maximum speed twice for 2 min intervals with a 3 min cooling period. A 250 µl aliquot of 0.15 M sodium chloride (0.9%) was added to the tube, vortexed for 2 min and allowed to stand 30 min. The samples were centrifuged for 3 min at 7800 x g before collection of the lower organic phase. Extracts were evaporated to dryness under nitrogen gas and resuspended in 500 µl of mobile phase A immediately prior to analysis. An Acquity liquid chromatography system employing a BEH C18 column (Acquity, 2.1 x 100 mm, 1.7 µm particle size) heated to 50°C, and a binary solvent system of 0.1% (v/v) methanol in water (mobile phase A) and acetonitrile/2-propanol (1:1 v/v) (mobile phase B) both containing 1 mM ammonium acetate, (pH 7.2) was used to resolve hepatic lipid extracts.([1](#_ENREF_1)) The samples were prepared by a 1:10 dilution in mobile phase A. A total of 5 µl was directly injected by the autosampler onto a 10 µl sample loop was employed. The chromatographic gradient consisted of ratios (A:B) of 65:35 (0-2 min), 20:80 (2-7 min), 0:100 (7-14 min), 65:35 (14-16 min) at a flow rate of 400 µl/min. Four injections per sample in positive ion mode were analyzed. Chromatographically-resolved lipids were analyzed by a hybrid quadrupole orthogonal time-of-flight mass spectrometer (SYNAPT, Waters, MS Technologies, Manchester, U.K.) in a manner similar to that previously published ([1](#_ENREF_1)). The mass spectrometer was operated in the MS^e^ mode when electrospray positive and negative ionization voltages were used. A capillary voltage of 2 kV, a cone voltage of 31V, an extraction cone voltage of 4 and a curtain gas flow rate of 21 L/min were used for both polarities. A desolvation source gas of 800 L/h at 350°C was used. Data acquisition took place over the mass range of 50-1200 Da. CHAPS (10 µg/ml; *m/z* 615.4037 in positive ion mode and *m/z* 659.3947 of CHAPS formate in negative ion mode) in acetonitrile:2-propanol:water (85:10:5, v/v/v) containing 10 mM ammonium formate, pH 4.0, was used as the lock mass introduced to the ionization source at 15 µl/min using an external pump (Harvard Apparatus, model C22). An integrated LockSpray unit with a reference sprayer automatically controlled by the acquisition software was used to collect a reference scan every 10 seconds lasting 0.1 second. During this acquisition method, the first quadrupole Q1 is operated in a wide band RF mode only, allowing all ions to enter the T-wave collision cell. Two discrete and independent interleaved acquisition functions were automatically created. The first function collected low energy unfragmented data while the second function collected high energy fragmentation data from a collision energy ramped from 15 to 40 eV for optimal signal responses (**Supplemental Figure S3**). In both instances, argon gas was used for CID. With this unbiased strategy both unfragmented and fragmented ions were consecutively monitored permitting quantification and species fragmentation, without prior knowledge of the sample composition. The latter experiment can be considered to be a product-ion scan, a precursor ion- or neutral-loss “like” scan. Automated quantification of acquired spectra was performed using MassLynx and MarkerLynx XS software (v4.2). Responses of internal standards within a concentration range of 10 ng to 250 ng (*R^2^* of 0.9592 for PC 17:0/14:1 and 0.9606 for PE 17:0-20:4) were linear when analyzed in positive mode (**Supplemental Figure S2).** Lipid peak intensities were normalized to internal standards and statistical comparisons made using ANOVA with a Bonferroni correction using Prism 5.04 (GraphPad Software, La Jolla, CA). Lipids were identified and annotated upon querying precursor and product ion masses against the Lipidmaps library (<http://www.lipidmaps.org>) as previously described ([1](#_ENREF_1)).

***Accurate Mass Reporting***

Lipids observed by MALDI IMS were identified by sub-parts per million (ppm) accuracy using an Apex-9.4-Qe FT-ICR (Bruker Daltonics, Bremen, Germany) equipped with a MALDI source, calibrated to ≤0.5 ppm accuracy. Tissue sections coated with sublimated DHB were acquired at a 200 µm lateral resolution for accurate mass profiling. The instrument was tuned to obtain high mass accuracy for broadband measurement over *m/z* 450-1600 with a 1 M transient (~ 200,000 resolution) averaging four scans per spectra with a flight time transient of 0.0016 seconds. The instrument was calibrated to ≤0.5 ppm accuracy using odd numbered red phosphorous clusters from P_15_-P_51_ (*m/z* 464.606971-1579.662386 (-), or 1579.661288 (+) ([2](#_ENREF_2)). Laser shot number and fluency was optimized for on-tissue signal using the abundant lipid phosphocholine 34:1, m/z 760.58508. Accurate mass determinations were made on at least 4 of the 8 samples per cohort.

1. Castro-Perez, J. M., J. Kamphorst, J. DeGroot, F. Lafeber, J. Goshawk, K. Yu, J. P. Shockcor, R. J. Vreeken, and T. Hankemeier. 2010. Comprehensive LC-MS E lipidomic analysis using a shotgun approach and its application to biomarker detection and identification in osteoarthritis patients. *J Proteome Res* **9**: 2377-2389.

2. Sladkova, K., J. Houska, and J. Havel. 2009. Laser desorption ionization of red phosphorus clusters and their use for mass calibration in time-of-flight mass spectrometry. *Rapid Commun Mass Spectrom* **23**: 3114-3118.
